# Phenylbutyrate Is Bacteriostatic against *Mycobacterium tuberculosis* and Regulates the Macrophage Response to Infection, Synergistically with 25-Hydroxy-Vitamin D₃

**DOI:** 10.1371/journal.ppat.1005007

**Published:** 2015-07-02

**Authors:** Anna K. Coussens, Robert J. Wilkinson, Adrian R. Martineau

**Affiliations:** 1 Clinical Infectious Diseases Research Initiative, Institute of Infectious Disease & Molecular Medicine, University of Cape Town, South Africa; 2 Division of Mycobacterial Research, MRC National Institute of Medical Research, London, United Kingdom; 3 Department of Medicine, Imperial College London, London, United Kingdom; 4 The Francis Crick Institute, Mill Hill Laboratory, London, United Kingdom; 5 Blizard Institute, Barts and The London School of Medicine, Queen Mary University of London, London, United Kingdom; New Jersey Medical School, UNITED STATES

## Abstract

Adjunctive vitamin D treatment for pulmonary tuberculosis enhances resolution of inflammation but has modest effects on bacterial clearance. Sodium 4-phenylbutyrate (PBA) is in clinical use for a range of conditions and has been shown to synergise with vitamin D metabolites to upregulate cathelicidin antimicrobial peptide (*CAMP*) expression. We investigated whether clinically attainable plasma concentrations of PBA (0.4-4mM) directly affect *Mycobacterium tuberculosis* (*Mtb*) growth and human macrophage and PBMC response to infection. We also tested the ability of PBA to enhance the immunomodulatory actions of the vitamin D metabolite 25(OH)D_3_ during infection and synergistically inhibit intracellular *Mtb* growth. PBA inhibited *Mtb* growth in broth with an MIC_99_ of 1mM, which was reduced to 0.25mM by lowering pH. During human macrophage infection, PBA treatment restricted *Mtb* uptake, phagocytic receptor expression and intracellular growth in a dose-dependent manner. PBA independently regulated CCL chemokine secretion and induced expression of the antimicrobial *LTF* (lactoferrin), the anti-inflammatory *PROC* (protein C) and multiple genes within the NLRP3 inflammasome pathway. PBA co-treatment with 25(OH)D_3_ synergistically modulated expression of numerous vitamin D-response genes, including *CAMP*, *CYP24A1*, *CXCL10* and *IL-37*. This synergistic effect was dependent on MAPK signalling, while the effect of PBA on *LTF*, *PROC* and *NLRP3* was MAPK-independent. During PBA and 25(OH)D_3_ co-treatment of human macrophages, in the absence of exogenous proteinase 3 (PR3) to activate cathelicidin, *Mtb* growth restriction was dominated by the effect of PBA, while the addition of PR3 enhanced growth restriction by 25(OH)D_3_ and PBA co-treatment. This suggests that PBA augments vitamin D–mediated cathelicidin-dependent *Mtb* growth restriction by human macrophages and independently induces antimicrobial and anti-inflammatory action. Therefore through both host-directed and bacterial-directed mechanisms PBA and vitamin D may prove an effective combinatorial adjunct therapy for tuberculosis to both resolve immunopathology and enhance bacterial clearance.

## Author Summary

Tuberculosis (TB) is the world’s leading bacterial cause of death. Effective treatment currently requires a minimum of 4 drugs taken for at least 6 months. While these drugs kill the TB causing bacteria (*Mtb*), they do not directly resolve the inmmunopathology associated with morbidity. Immunomodulatory agents that not only enhance an individual’s ability to kill *Mtb* but also help heal lung pathology could be used as adjuncts to current therapies to improve treatment outcome. Phenylbutyrate (PBA) has been in clinical use for more than 30 years to treat a range of conditions. It has also been shown to synergise with vitamin D to induce cellular production of the anti-*Mtb* peptide, cathelicidin. We investigated whether PBA and vitamin D synergistically kill *Mtb* in human macrophages and whether PBA has any independent effect on macrophages and *Mtb*. At concentrations that are achieved in plasma clinically, PBA inhibited *Mtb* growth. PBA also inhibited growth of *Mtb* in human macrophages via a cell-dependent mechanism, inducing the inflammasome pathway and antimicrobial lactoferrin. PBA also synergistically enhanced macrophage response to vitamin D and co-treatment further inhibited *Mtb* growth, when synergistically-induced cathelicidin was activated. PBA and vitamin D may therefore prove an effective combinatorial adjunct therapy for tuberculosis.

## Introduction

Tuberculosis (TB) is the world’s leading bacterial cause of death, with 9 million new cases and 1.5 million deaths in 2013 [1]. Despite the availability of antimicrobials, effective treatment regimes require long-term administration of multiple drugs, which can lead to toxicity, problems with adherence and the development of drug resistance. Current drug regimes, while reducing bacterial load, do not directly resolve the immunopathological inflammatory responses associated with morbidity [2, 3]. Immunomodulatory agents that augment antimicrobial activity and accelerate resolution of pulmonary inflammation could be used as adjuncts to antimicrobial therapy to improve treatment outcomes [4].

The active metabolite of vitamin D_3_, 1α25-dihydroxy-vitamin D_3_ [1α,25(OH)_2_D_3_] inhibits intracellular growth of *Mycobacterium tuberculosis* (*Mtb*) in peripheral blood mononuclear cells (PBMC) and monocytes (MN) and suppresses pro-inflammatory cytokine secretion *in vitro* [5, 6]. LL-37 (cathelicidin) has been identified as a mediator of vitamin D-dependent antimicrobial activity [7]. Pro-LL-37 (hCAP-18) is transcribed from the cathelicidin antimicrobial peptide (*CAMP;* OMIM 600474) gene that contains 3 vitamin D response elements (VDRE) in its promoter and requires enzymatic digestion by human neutrophil proteinase 3 (PR3; OMIM 177020) to produce mature LL-37 (OMIM 600474), an antimicrobial that directly inhibits *Mtb* growth [5, 8]. The presence of VDRE in the *CAMP* promoter is an evolutionary adaptation, having only been identified in chimpanzee and human DNA sequences [9]; as such the antimicrobial action of vitamin D mediated by *CAMP* is limited to higher-order primates. LL-37 also regulates autophagy-mediated killing via induction of autophagy related proteins Beclin-1 (OMIM 604378) and Atg5 (OMIM 604261) [10] and has chemotactic activity for polymorphonuclear leukocytes [11].

1α,25(OH)_2_D_3_ is synthesised from the major circulating form of vitamin D_3_, 25-hydroxy-vitamin D_3_ [25(OH)D_3_], by the vitamin D 1α-hydroxylase enzyme (CYP27B1; OMIM 609506) the expression of which is upregulated in leukocytes following ligation of toll-like receptors with *Mtb* ligands [12]. Vitamin D deficiency (defined as a circulating concentration <50nM 25(OH)D) is associated with susceptibility to tuberculosis [13, 14]. We have shown that administration of adjunctive vitamin D accelerates resolution of inflammatory responses during tuberculosis treatment [15], and that this is associated with a trend towards accelerated sputum culture conversion [16]. The limited *in vivo* effect of vitamin D supplementation on *Mtb* growth despite significant inhibition *in vitro* may be due to the fact that the majority of *in vitro* experiments have used supra-physiological doses of 1α,25(OH)_2_D_3_ (100nM *vs*. the circulating concentration of 100pM). Physiological doses (100nM) of 25(OH)D_3_ have only shown a significant effect on *Mtb* growth *in vitro* during co-stimulation with other molecules, such as IFNγ (OMIM 147570) or IL-15 (OMIM 600554), to prolong transcription of *CYP27B1* [6, 17, 18].

Sodium 4-phenylbutyrate (PBA) is an aromatic short chain fatty acid which has been in clinical use for more that 30 years. As a pro-drug for sodium phenylacetate it is used to control nitrogen disposal in urea disorders, as a chemical chaperone it is used to alleviate endoplasmic reticulum stress in type-2 diabetes, and as a histone deacetylase inhibitor (HDACi) it is used to treat various leukaemias and cancers via modulating chromatin structure and inducing cellular differentiation and apoptosis [19–24]. PBA has also been shown to have direct anti-fungal activity against *Candida albicans* and *Cryptococcus neoformans* [25]. Moreover, PBA synergises with the 1α,25(OH)_2_D_3_ induction of *CAMP* expression, in a variety of cell lines [26, 27]. This synergistic induction of *CAMP* expression was also recently confirmed *ex vivo* in PBMC from healthy control participants in a dose-finding study who received oral PBA and vitamin D_3_ for 8 days [28], also confirming the systemic effect of this treatment on immune cell function. On the basis of this synergistic *CAMP* induction, there is an on-going phase 2 trial of PBA and vitamin D_3_ adjunct therapy for TB [29]. However, the direct effect of PBA on *Mtb* and human immune cells is unknown. Data are also lacking on the question of whether this combined co-treatment has additional synergistic effects on macrophage function during *Mtb*-infection and if it can synergistically inhibit intracellular *Mtb* growth via *CAMP* induction. Thus, we investigated each of these questions, determining the effects of clinically attainable plasma concentrations of PBA (0.4-4mM), alone and in combination with the physiologically relevant vitamin D metabolite 25(OH)D_3_, at optimal concentration for immune function.

## Results

### PBA directly restricts *Mtb* growth

**Fig 1:**
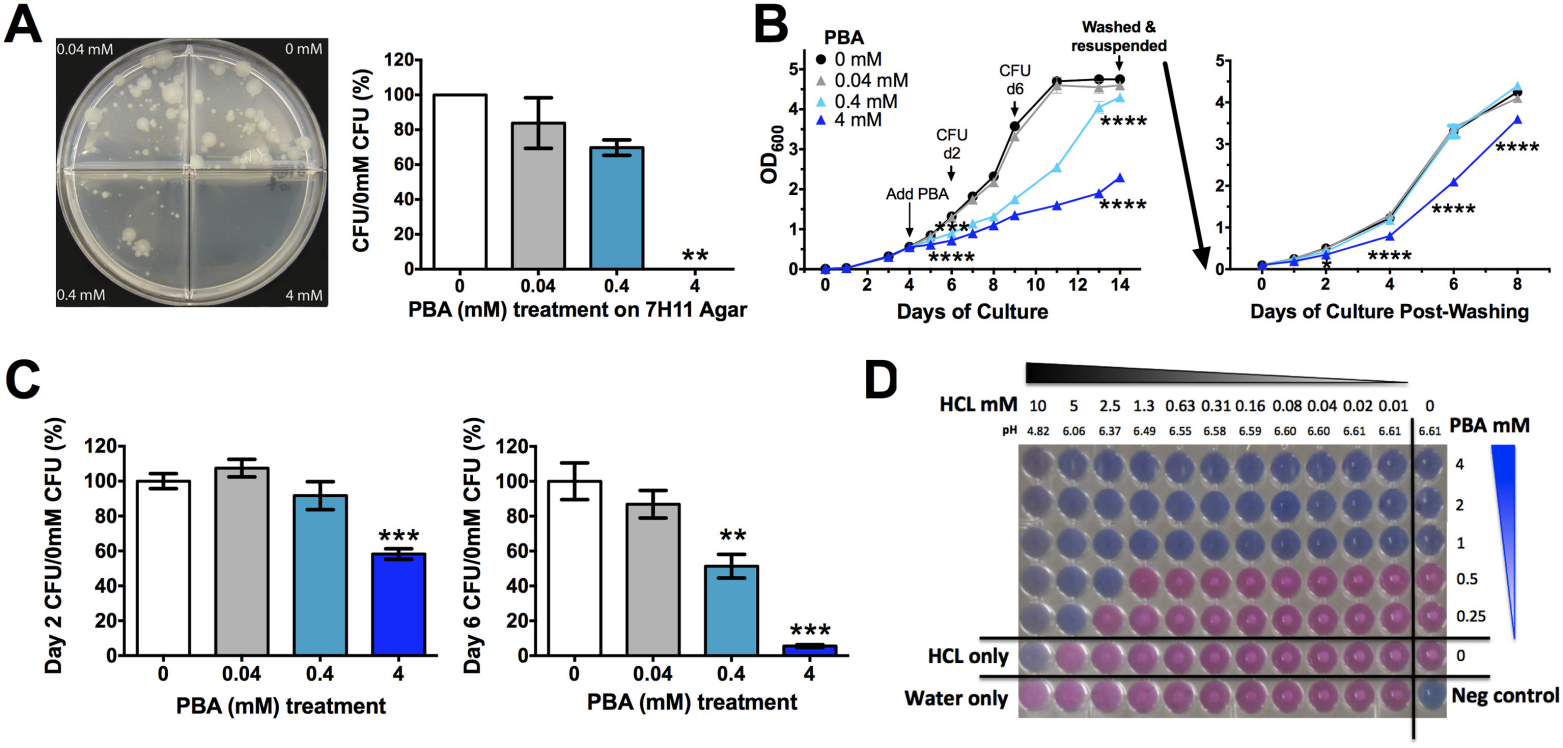
PBA inhihibts *Mtb* H37Rv growth on solid and in liquid medium. (A) *Mtb* 21 day growth on solid medium a 10-fold serial dilutin of PBA. (B) Growth of *Mtb* in rolling broth culutre as monitored by OD_600_ after addition of PBA on day 4 to a final concentration of 0.4-4mM PBA. 10-day treated culutres were washed and resuspended in PBA-free medium with growth monitored for a further 8 days. (C) *Mtb* growth in broth (B), assessed by CFU. (D) Checkerboard Alamar Blue reduction assay measuring MIC_99_ of PBA in the presence of 2-fold serial dilution of HCl, with pH of medium indicated. Growth occurs in pink wells with visible white *Mtb* pellets. Mean ± SEM, for dupicate experimetns, CFU plated in triplicate from growth curves, growth curves and Alamar Blue respresentative of replicate experiments, anlaysed by 1-way ANOVA, with Bonferroni multiple comparison test (A,C), or 2-way ANOVA, with Dunnett's multiple comparison test (B); *, P<0.05; **, P<0.01; ***, P<0.001, ****, P<0.0001.

To test whether PBA has direct anti-mycobacterial effects, *Mtb* was grown on both solid agar medium and in broth in the presence of a 10-fold serial dilution (0.4-4mM) of PBA. Plating a low density culture on solid medium containing 4mM PBA completely restricted growth, while the presence of 0.4mM PBA allowed 70% growth and 0.04mM allowed 84% growth, compared to vehicle control (0mM, 0.4% water) (Fig 1A). When PBA was added to exponentially growing rolling broth cultures, 4mM PBA significantly inhibited an increase in OD_600_ from day 2 onwards (P<0.0001) and this was maintained until day 14 at which stage cultures were pelleted, washed and resuspended in PBA-free medium (Fig 1B). Growth inhibition by 4mM PBA was maintained in PBA-free medium for a further 8 days and growth never reached control levels (P<0.0001) (Fig 1B). Reductions in OD_600_ were confirmed by colony forming unit (CFU) analysis, with 4mM PBA reducing CFU to 6% of control after 6 days of treatment (Fig 1C). The retardation of growth following removal of 4mM PBA from the medium suggests that PBA is bacteriostatic rather than bactericidal and it may modify bacterial replication.

Phenotypically when exponential phase cultures were grown in the presence of 4mM PBA, even in the presence of a detergent, the cultures formed a cord like conglomerate that was easily dispersed by swirling. This suggested that there may be a cell wall modification by PBA, resulting in the bacteria having an adherent phenotype. Confocal microscopy of GFP-expressing H37Rv confirmed that PBA-treated bacilli are often found bound along the entire shaft of neighbouring bacilli, forming lined-up clusters (S1A Fig), unlike untreated cultures which had single bacilli or overlapping bacilli when in clusters (S1B Fig). Non GFP-expressing bacteria were also found within these PBA clusters of lined-up bacilli indicating that they were not metabolically active (S1A Fig).

As a weak acid, PBA activity may be modulated by the pH of the growth medium. We confirmed that there was no significant effect of PBA on pH of growth medium from infected and uninfected cultures (S2 Fig). We then tested a 2-fold serial pH reduction over a range of PBA concentrations (0.25–4mM) using the Alamar Blue reduction checkerboard assay. The minimum inhibitory concentration of PBA which stopped 99% of *Mtb* growth (MIC_99_) without HCl was 1mM and this was reduced to 0.5mM in the presence of 2.5mM HCl and to 0.25mM in the presence of 5mM HCl, with 10mM HCl completely inhibiting *Mtb* growth irrespective of PBA (Fig 1D). At these HCl concentrations growth medium pH reduced from 6.61 to 6.37, 6.06 and 4.82, respectively (Fig 1D). *Mtb* containing phagosomes have a pH range of 6.5 to 6.2 and become acidified to pH 5.8 to 5.6 when infected with attenuated *Mtb*, and can reach a pH of 5.0 to 4.5 upon phagolysosome fusion [30, 31]. Thus, PBA may have enhanced activity against *Mtb* as phagosomes become increasingly acidified.

### PBA restricts *Mtb* uptake and intracellular growth by PBMC and macrophages

**Fig 2:**
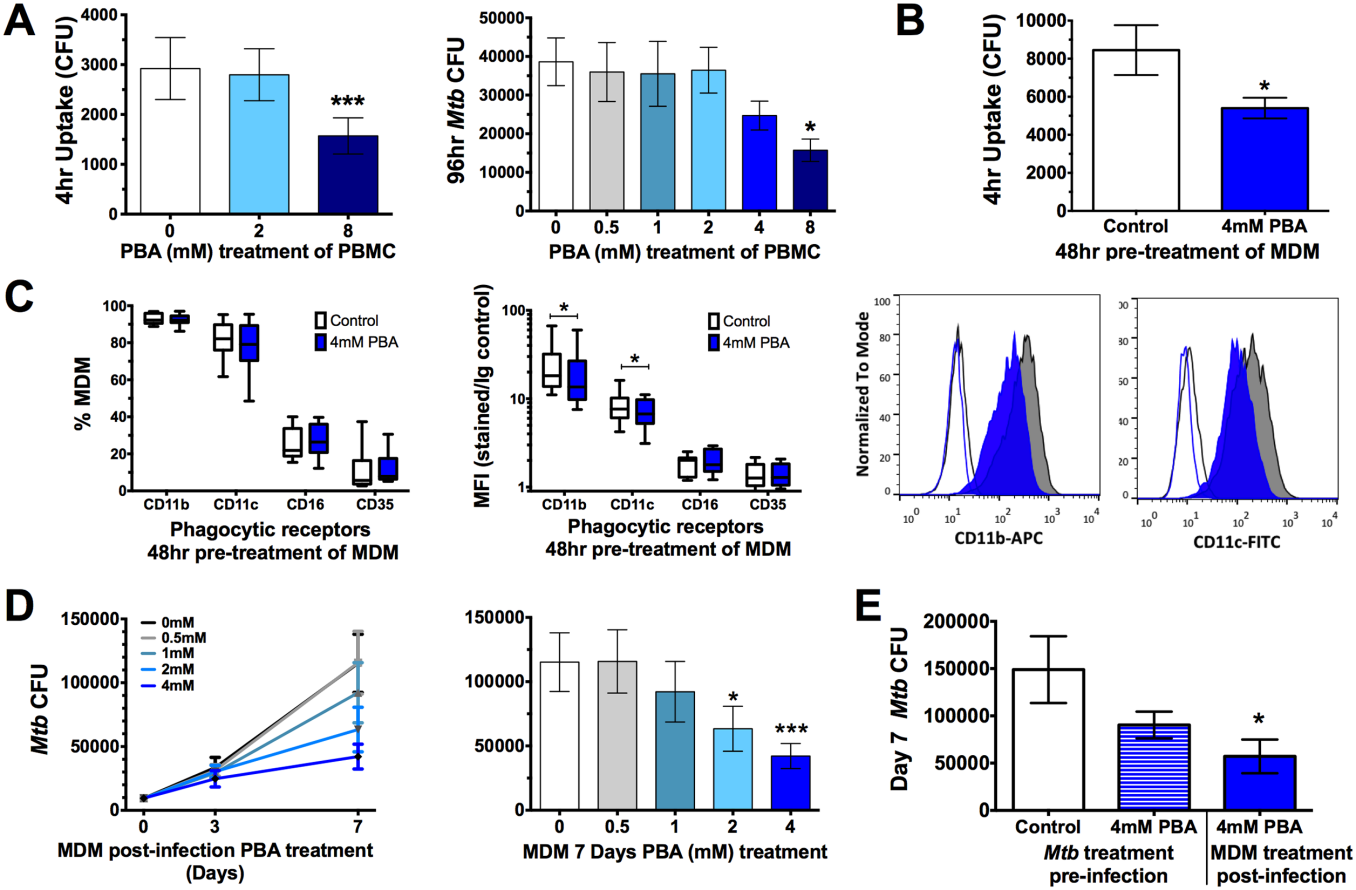
PBA resticts *Mtb* uptake and intracellular growth in PBMC and MDM. (A) Uptake of *Mtb* H37Rv by PBMC 4 hrs post-infection and intracellular growth 96 hr-post-infection in the presence of a 2-fold serial dilution of PBA. (B) *Mtb* uptake by MDM pre-treated with 4mM PBA for 48 hrs. (C) Phagocytic receptor expression (CD11b, CD11c, CD16 or CD35) on MDM following 48 hrs 4mM PBA treatment:percent of live cells expressing receptors and median fluorescent index (MFI), by flow cytometry. (D) Intracellular *Mtb* growth in MDM treated with a serial dilution of PBA 4 hrs post-infection, measured by CFU. (E) Seven day intracellular growth of 4mM PBA pre-treated *Mtb*, and control *Mtb*, grown in MDM ± 4mM PBA post-infection. Mean ± SD or min-max (box plots), n = 3–4 in triplicate for CFU and n = 8 for flow cytometry; 1-way ANOVA with Dunnett’s multiple comparisons test (A,E,F) or Paired t-test (B,C,D): *, P<0.05, ***, P<0.001.

Once we had determined PBA had a direct effect on *Mtb*, we next investigated the effect of PBA on the human cellular response to *Mtb*. Initially, a 2-fold serial dilution of PBA (0.5-8mM) was added to PBMC at the time of *Mtb* infection. Uptake was significantly inhibited 4 hrs post-infection by 8mM PBA (to 54% of control, P = 0.0009) and intracellular growth was inhibited after 96 hrs in PBMC, in a dose-dependent manner: there with a trend for decreased growth with 4mM PBA (71% of control) and a significant inhibition of growth with 8mM PBA (45% of control, P = 0.01) (Fig 2A).

The inhibition of *Mtb* uptake when PBA was added at the time of infection suggests PBA may directly affect phagocytic function. To confirm this, monocyte-derived macrophages (MDM) were pre-treated for 48 hrs with PBA, washed and then infected with *Mtb* in PBA-free medium. Pre-treatment significantly inhibited MDM uptake of *Mtb* to 64% of control (P = 0.02, Fig 2B). To determine if this was associated with inhibited expression of phagocytic receptors, macrophages were analysed for surface expression of CD35 (CR1, OMIM 120620), CD11b (CR3, OMIM 120980), CD11c (CR4, OMIM 151510), and CD16 (FcγR, OMIM 146740) by flow cytometry. PBA pre-treatment did not affect the number of MDM expressing each receptor but it did significantly inhibit median fluorescent index of CD11b and CD11c (Fig 2C).

The effect of PBA on *Mtb* intracellular growth in MDM was subsequently assessed by treating MDM with PBA 4 hrs post-infection, to ensure comparable infection of cells pre-treatment. There was a trend for decreased intracellular growth with 4mM PBA (74%) after 72 hrs of infection, while 2-4mM PBA restricted growth after 7 days (to 55% and 37% of control, respectively, P<0.012, Fig 2D). To elucidate whether *Mtb* growth inhibition was mediated via a cellular-dependent mechanism or if it was due to the direct effect of PBA on *Mtb* growth, *Mtb* was cultured for 4 days in the presence/absence of 4mM PBA, extensively washed to remove PBA and then MDM were infected for 4 hrs and cultured in medium without PBA for 7 days. CFU analysis indicated that there was no significant effect of PBA pre-treatment of *Mtb* on infection dose or 4 hrs uptake (S3 Fig) and only a small non-significant reduction in *Mtb* growth after 7 days (Fig 2E). Conversely when MDM from the same donors were infected with untreated *Mtb* and MDM were treated post-infection with 4mM PBA *Mtb* growth was significantly inhibited (P = 0.018, Fig 2E). This suggests that PBA also has a direct effect on cell-mediated growth restriction and that the ability of PBA to restrict the growth of intra-phagosomal bacteria is more likely mediated *via* a host-directed mechanism than *via* the direct action of PBA.

### PBA regulates macrophage transcriptional response to *Mtb* infection

**Fig 3:**
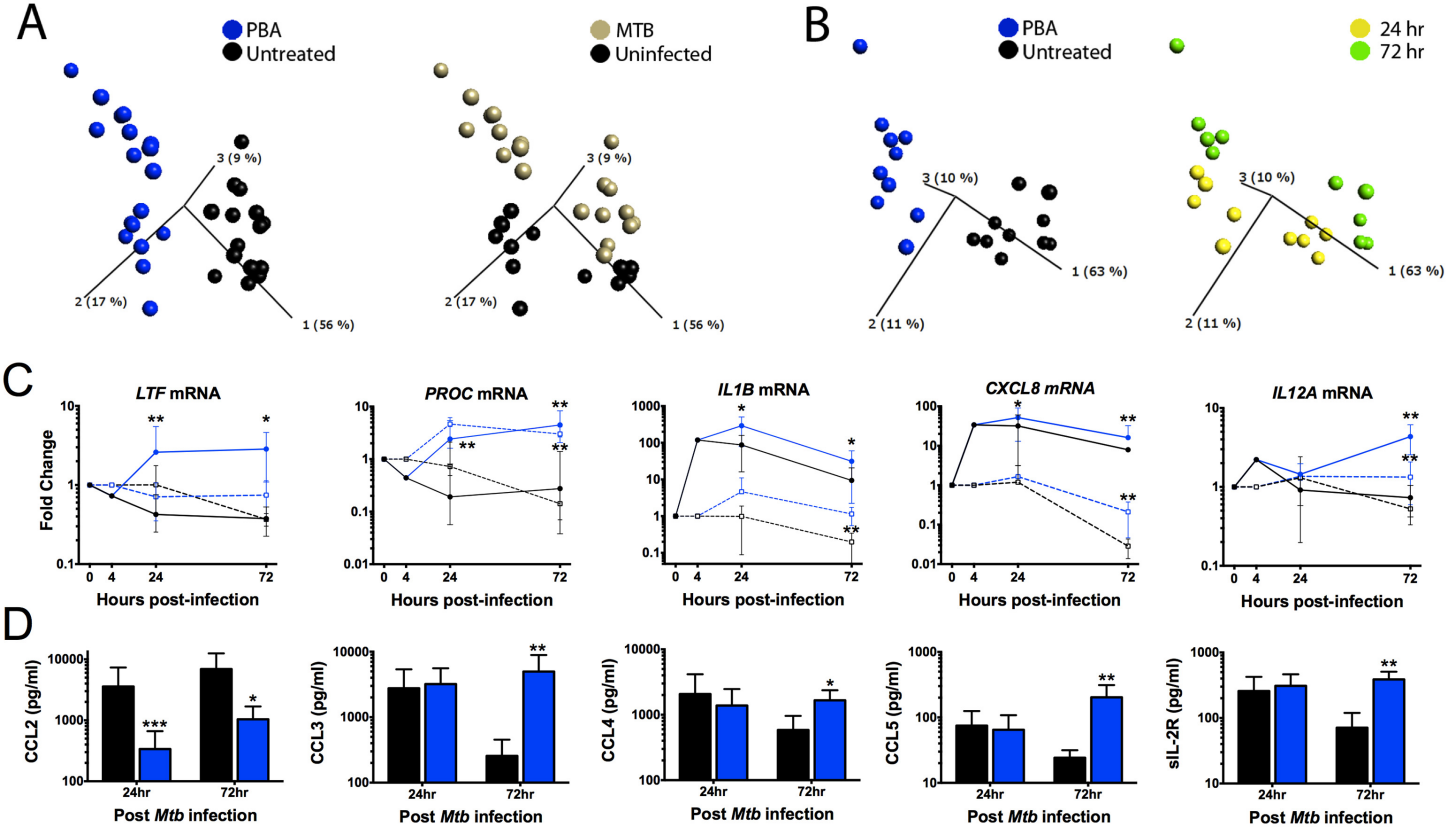
PBA modulates gene transcription by macrophages, irrespective of *Mtb* infection. (A) Principal component anlaysis (PCA) of the 12 genes significantly regulated by PBA, irrespective of time and infection status. Each point represents one donor and condition, and its position in the plot is determined by the combined effects of all 12 genes for that treatment, time and infection type. The distance between points represents Euclidean distance. The first 3 component vectors are displayed, along with a % figure signifying the proportion of the variability in the data that each component accounts for. Points are coloured according to treatment type (left) or infection status (right). (B) PCA analysis of *Mtb*-infected cells coloured by treatment (left) or time of sampling (right). (C) Pattern of gene expression of 5 genes regualted by PBA (blue) vs control (black) in *MTb*-infected (circles, solid-lines) and uninfected MDM (squares, dashed-lines). (D) Protein secretion by MDM 72hr post-*Mtb* infection. Mean ± SD, n = 4–5; P-values from t-test for GLM, with adjustment for donor variation (Table 1); *, P<0.05; **, P<0.01.

To investigate how PBA regulates macrophage response to *Mtb* infection, MDM gene expression was analysed using real-time reverse transcription-polymerase chain reaction (RT-PCR) arrays designed for a panel of 28 immune response and vitamin D-associated genes. Initially, to gain a broad sense of the transcriptional regulation by PBA, expression results from infected and uninfected MDM at 24 and 72 hrs post-infection (20 and 68 hrs post-treatment) were combined for analysis, applying a t-test for general linear models (GLM) with statistical adjustment for donor variation and time of treatment. Principal component analysis (PCA) conducted using the 12 genes identified to be significantly regulated by PBA, irrespective of time and infection status, showed that PBA had the greatest effect on gene expression in infected cells (Fig 3A). We then applied a similar GLM analysis to data from only infected cells, and found PBA had a greater effect on gene expression after 72 hrs (Fig 3B).

Gene expression data were then analysed separating samples from each time point and infection status, to identify all genes regulated by PBA under the different conditions (Table 1). PBA only affected uninfected cells after 72 hrs: seven genes were upregulated, six of which were also upregulated during *Mtb* infection (*CAMP*, *CXCL8* (OMIM 146930), *IL1B* (OMIM 147720), *IL12A* (OMIM 161560), *PROC* (OMIM 176860), *VDR* (OMIM 601769)) and one gene was inhibited (*CYP27B1*).

**Table 1:** Effect of 4mM PBA on MDM gene expression and protein secretion.

		*Mtb*-infected				Uninfected	
		24 hrs		72 hrs		72 hrs^1^	
	Analyte	Fold change^2^	*P*	Fold change	*P*	Fold change	*P*
**mRNA**	PROC	15.31	0.0015	20.14	0.0096	23.33	0.0017
	LTF	6.78	0.0079	6.23	0.0225	-	ns
	IL36G	5.52	0.0213	6.62	0.0229	-	ns
	IL1B	4.29	0.0116	3.82	0.0252	6.56	0.0036
	IL37	3.80	0.0028	5.26	0.0099	-	ns
	IL23A	2.84	0.0094	-	ns	-	ns
	CXCL8	1.92	0.0461	3.24	0.0090	6.92	0.0045
	TREM1	1.85	0.0453	2.82	0.0427	-	ns
	CASP1	1.81	0.0001	2.12	0.0487	-	ns
	NCF4	1.53	0.0042	-	ns	-	ns
	ARG1	-1.45	0.0085	-	ns	-	ns
	CYP27B1	-1.61	0.0347	-	ns	-6.25	0.0005
	IL10	-2.74	0.0208	-	ns	-	ns
	CXCL7	-4.03	0.0104	-	ns	-	ns
	IL12A	-	ns	6.21	0.0093	2.35	0.0067
	CAMP	-	ns	3.40	0.0030	3.90	0.0189
	NLRP3	-	ns	2.44	0.0263	-	ns
	VDR	-	ns	2.14	0.0121	2.42	0.0096
	IFNB1	-	ns	-	ns	1.72	0.0168
**Protein**	CCL3	-	ns	19.73	0.0032		
	CCL5	-	ns	6.91	0.0023		
	IL-2R	-	ns	5.99	0.0018		
	CCL4	-	ns	3.16	0.0363		
	CCL2	-10.54	9.17x10^-5^	-5.78	0.0269		

^1^Uninfected mRNA had no genes significant affected by PBA at 24 hrs (q≤0.1).

^2^Fold change and P-value from t-test for GLM with adjustment for subject ID (repeated measures, n = 5), on samples from the specified time point and infection status, non-significant (ns) if false discovery rate (q-value) >0.1

During *Mtb* infection, 18 genes were regulated by PBA: 10 genes were induced at 24 hrs and 12 genes at 72hrs, with eight genes induced at both time points (*CASP1* (OMIM 147678), *CXCL8*, *IL1B*, *IL36G* (OMIM 605542), *IL37*(OMIM 605510), *LTF* (OMIM 150210), *PROC*, *TREM1* (OMIM 605085)), while four genes were inhibited and only at 24 hrs (*ARG1* (OMIM 608313), *CXCL7* (OMIM 121010), *CYP27B1*, *IL10* (OMIM 124092)) (Table 1). The effect of PBA on gene expression was also dose-dependent (S4 Fig). However, while PBA had a greater effect on the number of genes regulated when treatment occurred during infection, the fold change in expression regulated by PBA was actually higher in uninfected cells (Table 1), suggesting that *Mtb*-infection interferes with the effect of PBA treatment.

The 2 genes most highly induced by PBA during infection, were *LTF* and *PROC*. *LTF* encodes lactoferrin, an iron chelator with known anti-*Mtb* effects [32], which PBA induced approximately 6-fold (P<0.022), the effect of PBA being infection-dependent (Fig 3C). *PROC*, which encodes anticoagulant protein C, an anti-inflammatory molecule which supresses cytokine production and secretion [33] had the greatest induction (15–23 fold, P<0.01), irrespective of infection (Fig 3C and Table 1). *CAMP* expression was also induced, but only 3-4-fold (P≤0.019) 72 hrs post-treatment (Table 1).

Of the 8 genes regulated by PBA during *Mtb* infection at both time points, four were involved in the inflammasome and IL-1 pathway; *IL1B*, *IL36G* (*IL1F9*), *IL37* (*IL1F7b*) and *CASP1* (required for IL-1β post-translational activation) were induced. *NCF4* (OMIM 601488) which transcribes a subunit (p40-phox) of NAPDH oxidase which activates the NLRP3 inflammasome, and *NLRP3* (OMIM 606416) were both induced by PBA during infection, at 24 and 72 hrs, respectively (P≤0.026). Six additional *Mtb*-associated genes encoding cytokines and chemokines were also regulated by PBA. Of these, *CXCL8* (IL-8) and *IL12A* (IL-12p35) had the greatest induction of expression (3-6-fold, P = 0.009) (Fig 3C and Table 1).

### PBA regulates macrophage chemokine secretion during *Mtb* infection

The effect of PBA on macrophage secretion of a panel of 30 chemokines, cytokines and growth factors in supernatants 24 and 72 hrs post-infection was then analysed, using a t-test for GLM with adjustment for donor variation. Similar to the effects on transcript abundance, PBA had a greater effect on protein secretion 72 hrs post-infection, modulating secretion of five analytes, compared to only one at 24 hrs (Fig 3D and Table 1). A dose-response was also confirmed for these proteins (S5 Fig). Four of the five analytes were C-C ligand (CCL) chemokines: CCL2 (OMIM 158105) secretion was inhibited by PBA at 24 and 72 hrs (P≤0.027), while CCL3 (OMIM 182283), CCL4 (OMIM 182284) and CCL5 (OMIM 187011) had increased secretion after 72 hrs (Fig 3D, P≤0.036). The concentration of soluble IL-2R (OMIM 146710) was also significantly higher in PBA treated cultures at 72 hrs (P = 0.0018).

### PBA and 25(OH)D_3_ synergistically and independently modulate MDM immune response to *Mtb*

Since PBA had a significant effect on macrophage gene expression and protein secretion during *Mtb* infection, we next investigated the hypothesis that PBA synergises with 25(OH)D_3_ regulated gene expression during *Mtb* infection. Dose response experiments showed 100nM 25(OH)D_3_ was the optimal physiological concentration for transcriptional activation (serum levels ≥ 220nM are considered toxic [34]) (S6 Fig). MDM were therefore treated 4 hrs post-infection with 4mM PBA, 100nM 25(OH)D_3_ or in combination and RNA was isolated 24 and 72 hrs post-infection for analysis by RT-PCR arrays, as before. To identify the synergistic effects of PBA and 25(OH)D_3_ during co-treatment, a t-test for GLM, with adjustment for donor variation and time of treatment, was applied to three comparisons: 25(OH)D_3_ *vs*. control, co-treatment *vs*. control, and co-treatment *vs*. PBA (to determine the effect of 25(OH)D_3_ in the presence of PBA), including samples from both time points. A total of 20 genes were identified to be significantly regulated in at least one of the three treatment conditions (Table 2).

**Table 2:** Effect of 25(OH)D and PBA co-treatment on *Mtb*-infected MDM gene expression and protein secretion.

		25(OH)D vs Control		25(OH)D+PBA vs Control		25(OH)D+PBA vs PBA	
	Analyte	Fold change^1^	*P*	Fold change^1^	*P*	Fold change^1^	*P*
**mRNA**	CYP24A1	2922.85	2.29 x10^-7^	9740.72	6.29 x10^-10^	4260.58	5.94 x10^-9^
	CAMP	54.01	0.0002	195.63	0.0003	105.96	9.70 x10^-5^
	IL37	12.6	2.41 x10^-5^	35.91	1.22 x10^-6^	7.25	5.00 x10^-5^
	IL36G	8.85	0.0014	14.4	0.0009	2.84	0.0222
	TREM1	5.27	8.54 x10^-5^	6.92	1.26 x10^-5^	3.98	0.0002
	NOD2	2.45	0.0049	-	ns	-	ns
	PROC	-	ns	9.09	1.24 x10^-5^	-3.74	0.0067
	ARG1	-	ns	3.62	0.0384	2.22	0.0342
	LTF	-	ns	3.45	0.0075	-	ns
	IL1B	-	ns	3.19	0.0395	-	ns
	IL12A	-	ns	-	ns	-3.86	0.0109
	IL10	-	ns	-1.56	0.0372	-	ns
	IFNG	-	ns	-3.24	0.0563	-	ns
	VDR	-2.09	0.0036	-1.70	0.0152	-2.35	0.0011
	TNF	-2.56	0.0136	-2.04	0.0571	-	ns
	CXCL9	-4.16	0.0011	-8.94	0.0394	-	ns
	CYP27B1	-6.15	0.0033	-3.17	0.001	-2.56	0.0022
	CASP1	-5.78	1.70 x10^-5^	-	ns	-3.00	0.001
	CXCL7	-8.76	0.0015	-25.67	0.0001	-3.96	0.0062
	CXCL10	-9.54	0.0031	-33.02	0.0027	-10.67	0.0162
**Protein**	IL-1B	2.84	0.0012	-	ns	-	ns
	CXCL9	-	ns	-2.31	0.0299	-	ns
	CXCL10	-1.69	0.0172	-2.43	0.0027	-2.63	0.0045
	CCL2	-5.00	0.0004	-23.13	0.0001	-2.77	0.0156
	CCL5	-	ns	-	ns	-2.21	0.0157

^1^Fold change and p-value from t-test for GLM on indicated samples at 24 and 72 hrs with adjustment for subject ID (repeated measures, n = 3–4) and time; non-significant (ns) if false discovery rate (q-value) >0.1

**Fig 4:**
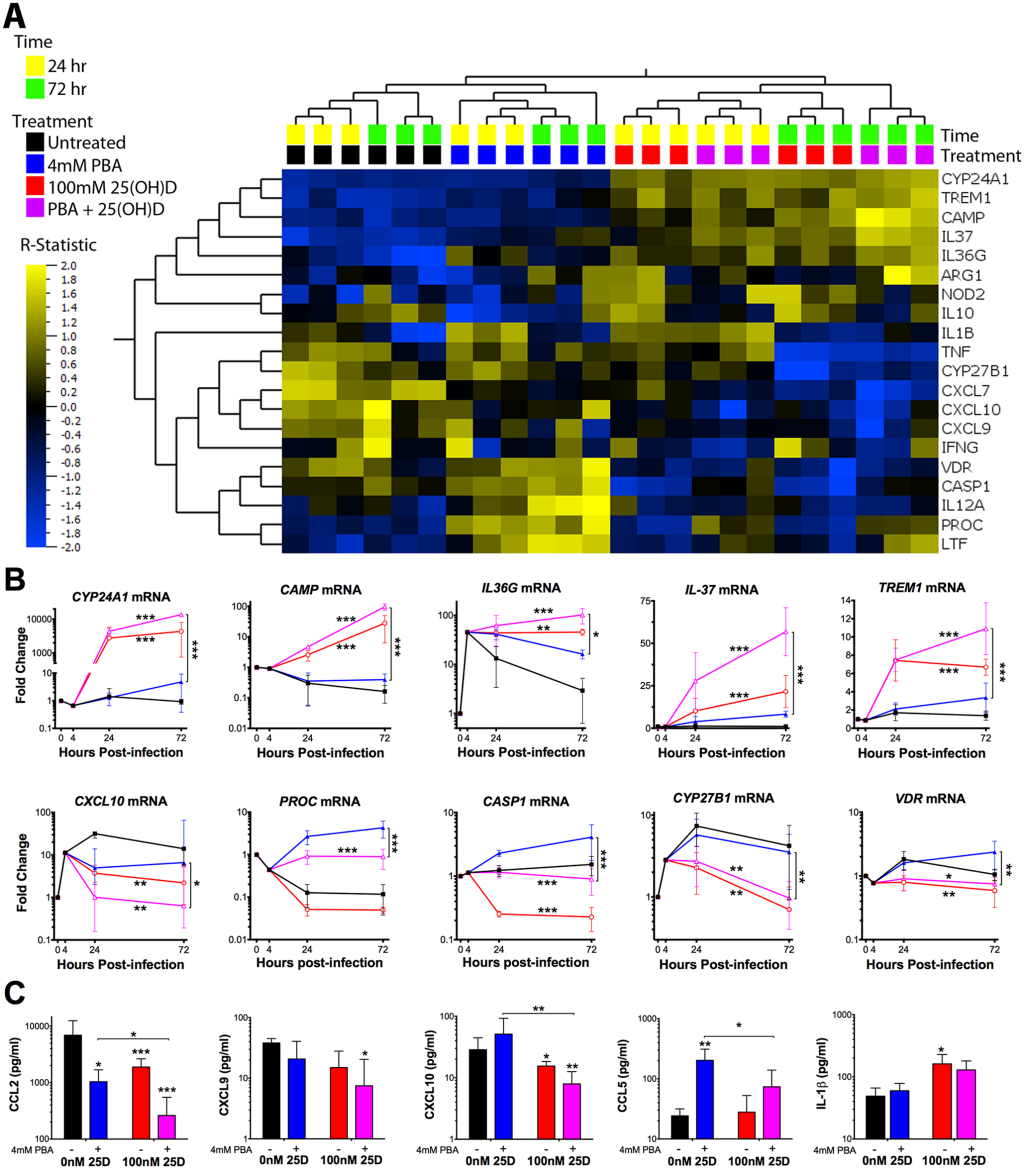
PBA and 25(OH)D_3_ synergistically and independently modulate MDM response to *Mtb* infection. (A) Heirachical clustering of the 20 genes identified to be differentially regulated by at least one of the three comparisons: 25(OH)D_3_ (red) vs control (black), co-treatment (purple) vs control (black) and co-treatment vs PBA (blue) (Table 2). Gene expression of 25(OH)D_3_ treated cells clusteres with co-treated samples, while PBA-treated samples cluster seperately. The patterns of expression also cluster according to time of sampling, with the R-statistic, representing the degree of difference for each comparison. Heirachcal clustering of genes (left hand tree dendogram) displays the correlation between regualted genes. (B) MDM gene expression during *Mtb*-infection over 72 hours. (C) MDM protein secretion 72 hrs post-infection. Mean ± SD, n = 3–4; P-values from t-test for GLM, with adjustment for donor variation and time of treatment with false discrovery rate q<0.1 (Tables 1 and 2); *, P<0.05; **, P<0.01; ***, P<0.001.

Hierarchical clustering applied to the list of 20 differentially regulated genes showed that 25(OH)D_3_- and co-treated samples clustered together, indicating that 25(OH)D_3_ had the greatest contribution to differential gene expression during co-treatment. PBA-treated samples formed their own cluster and all treatments clustered according to time of sampling, with 72 hrs samples showing a greater effect of each treatment (Fig 4A).

Of the 13 genes regulated by 25(OH)D_3_, six were also regulated by PBA monotherapy (although the effect of 25(OH)D_3_ on their expression was greater: four were induced [*CAMP*, *IL36*, *IL37* and *TREM1*] and two were inhibited [*CXCL7*, *CYP27B1*]), two were regulated in an opposite direction to PBA (*CASP1* and *VDR*) and four (*CXCL9* (OMIM 601704), *CXCL10* (OMIM 147310), *NOD2* (OMIM 605956), *TNF* (OMIM 191160)) were not significantly regulated by PBA monotherapy (Tables 1 and 2). Adding 25(OH)D_3_ in the presence of PBA enhanced the response of eight 25(OH)D_3_-regulated genes: five (*CAMP*, *CYP24A1* (OMIM 126065), *IL36*, *IL37*, *TREM1*) were synergistically induced, with the combined effect of co-treatment greater than an additive combination of individual effects, and three chemokines *CXCL7*, and IFNγ-inducible *CXCL9* and *CXCL10* were all further inhibited by combined treatment, despite the fact that PBA only showed a trend towards inhibition of *CXCL9* and *CXCL10* during monotherapy (Fig 4B and Table 2). *IFNG* was also inhibited by co-treatment, but there was no independent effect of PBA or 25(OH)D_3_ on *IFNG* expression (Tables 1 and 2). We also analysed the effect of co-treatment on uninfected cells and near identical patterns of synergism were observed for induced genes, except for the CXC chemokines which were only inhibited during infection (S7 Fig).

Of the two genes which were differentially regulated by PBA and 25(OH)D_3_ monotherapies, *CASP1* showed an intermediate expression between the two therapies during co-treatment with the effect dominated by 25(OH)D_3_, such that expression of *CASP1* was inhibited during co-treatment. Furthermore, 25(OH)D_3_ attenuated *PROC* induction by PBA, despite not having a significant effect of *PROC* during monotherapy (Fig 4B and Table 2).

To determine whether the synergistic effect of PBA on 25(OH)D_3_ gene expression was potentially associated with increased 1,25(OH)D_3_ production or activity, the expression of *CYP27B1* and *VDR* was also assessed, respectively. The inhibition of *CYP27B1* and *VDR* expression by 25(OH)D_3_ was very moderately attenuated by co-treatment with PBA (Fig 4B and Table 2), suggesting PBA may enhance the response to 25(OH)D_3_ through increased VDR signalling, particularly as PBA monotherapy induces *VDR*.

### PBA and 25(OH)D_3_ have limited synergistic effect on cytokine and chemokine secretion

We next investigated whether co-treatment with PBA and 25(OH)D_3_ had a synergistic effect on cytokine/chemokine secretion in supernatants from the same *Mtb*-infected MDM by applying the same GLM analysis technique to secretion data as for expression data. Only three analytes of the 30 investigated were identified to be significantly regulated by 25(OH)D_3_ and only three were regulated during co-treatment (Table 2). CCL2 secretion, which was significantly inhibited by PBA (Fig 3D), was also inhibited by 25(OH)D_3_ and synergistically inhibited during co-treatment (Fig 4C). Reflecting gene expression results, secretion of CXCL9 and CXCL10 was inhibited during co-treatment despite only CXCL10 being inhibited by 25(OH)D_3_ monotherapy, while neither was inhibited by PBA monotherapy. Conversely, an opposing effect of co-treatment was observed for CCL5 and IL-1β, 25(OH)D_3_ attenuated CCL5 induction by PBA, while PBA attenuated 25(OH)D_3_ induction of IL-1β (Fig 4C, Table 2).

### PBA and 25(OH)D_3_ influence transcript abundance via different pathways

**Fig 5:**
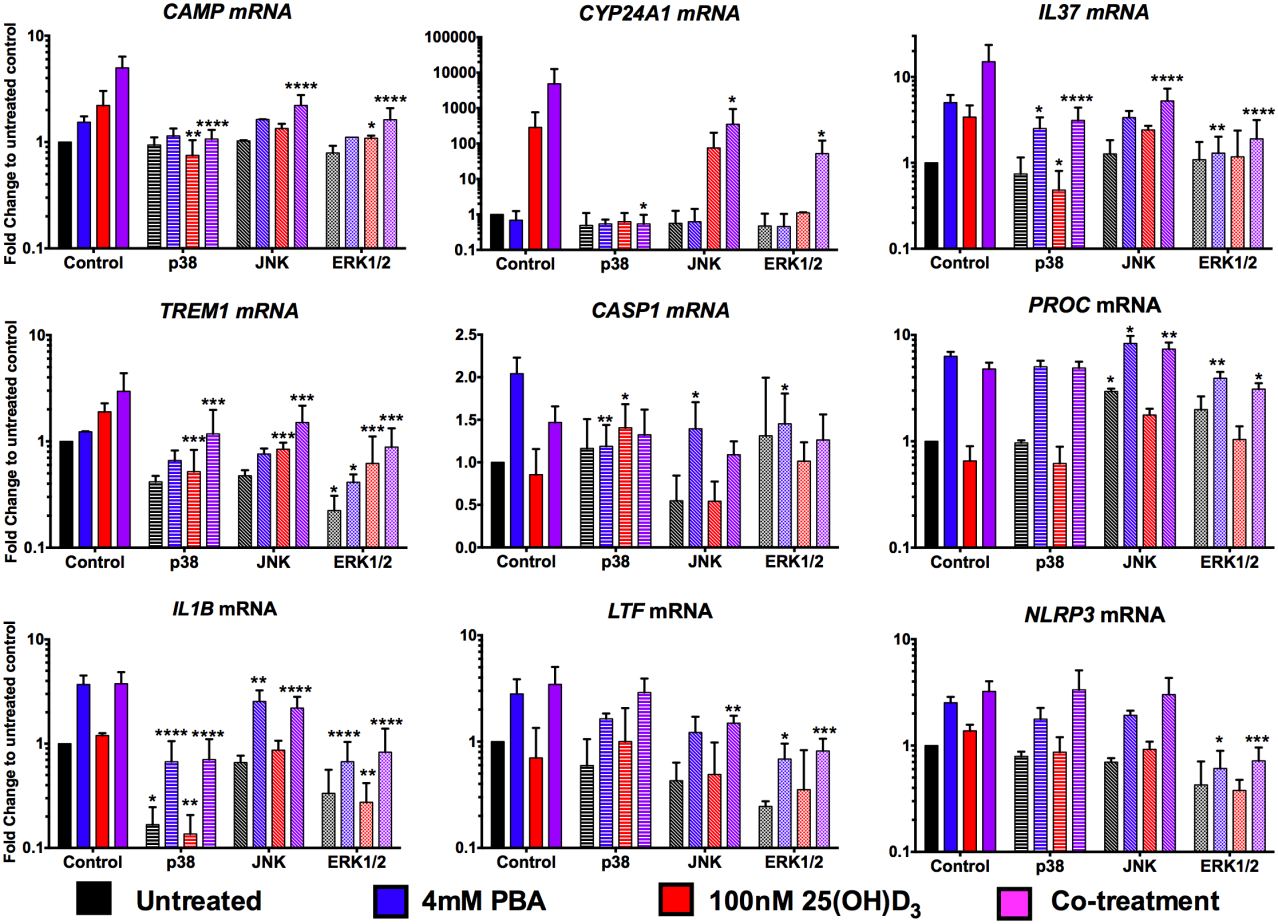
PBA synergistically regulates 25(OH)D_3_-mediated gene induction via MAPK signalling pathways. MDM were incubated for 1hr with 20μM of MAPK signalling inhibitors for p38 (SB202190), JNK (SP600125) and ERK1/2 (U0126) pathways, prior to addition of 4mM PBA (blue), 100nM 25(OH)D_3_ (red) or co-treatment (purple) and gene expression analysed 24 hrs post-treatment. Mean ± SD, n = 3–4; 2-way ANOVA with Dunnett’s multiple comparisons test: *, P<0.05; **, P<0.01; ***, P<0.001; ****, P<0.0001.

In an epithelial cell line, mitogen-activated protein kinase (MAPK) signalling via JNK (OMIM 601158) and ERK1/2 (OMIM 601795/176948) pathways has been shown to regulate PBA-induced *CAMP* expression [26]. We therefore investigated whether PBA regulated synergistic 25(OH)D_3_ gene expression in macrophages via the same MAPK pathways and whether it also utilised these pathways to regulate non-vitamin D-associated genes. MAPK pathway inhibitors for p38 kinase (SB202190), JNK (SP600125) and ERK1/2 (U0126) were added to MDM 4 hrs post-infection, for 1 hr prior to addition of PBA ± 25(OH)D_3_ and gene expression analysed 24 hrs post-treatment (Fig 5). Synergistic induction of *CAMP* and *CYP24A1* was completely inhibited by p38 kinase inhibition, and only partially by ERK1/2 and JNK inhibition. P38 kinase inhibition also had the greatest effect on attenuating *IL37* induction by 25(OH)D_3_, while ERK1/2 inhibition had the greatest effect on attenuating PBA and co-treatment induction of *IL37*. However, for *TREM1*, while p38 inhibition prevented induction by 25(OH)D_3_ and ERK1/2 inhibition somewhat attenuated PBA, ERK1/2 inhibition also attenuated expression in control cells and none of the MAPK inhibitors prevented synergistic induction, compared to control.

Analysis of the two genes differentially regulated by PBA and 25(OH)D_3_, *CASP1* and *PROC*, indicated very different patterns for the two treatments: *CASP1* induction by PBA was attenuated by all three pathways although the most via p38, while p38 also attenuated *CASP1* inhibition by 25(OH)D_3_. Conversely, *PROC* expression was only moderately suppressed by ERK1/2 inhibition, during PBA and co-treatment, again not to untreated levels, and JNK inhibition actually led to increased expression by PBA and co-treatment (Fig 5).

Finally, when we investigated genes independently regulated by PBA, we found that even though *IL1B*, *LTF* and *NLRP3* expression can be inhibited via at least one of the signalling pathways, the induction by PBA of *IL1B* and *LTF* occurred even when these pathways were suppressed and only *NLRP3* induction was partially attenuated by inhibition of ERK signalling (Fig 5). These results indicate that the vitamin D-independent mechanism of PBA-regulated gene expression is predominantly mediated via pathways other than MAPK signalling.

### 25(OH)D_3_ and PBA have independent and additive anti-mycobacterial effects

**Fig 6:**
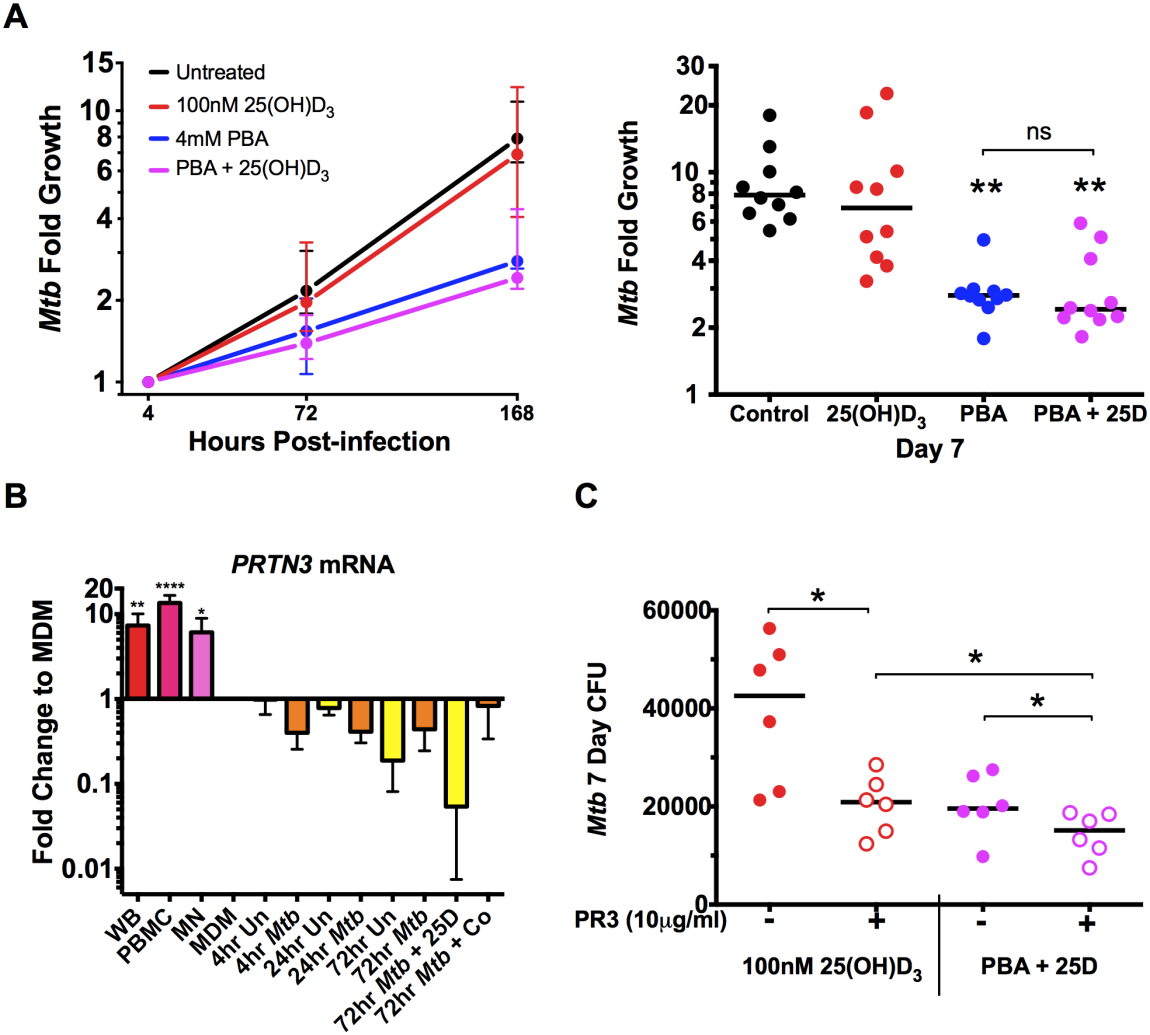
PBA restricts intracellular *Mtb* growth independently of 25(OH)D_3_. (A) Intracellular growth of *Mtb* in MDM treated 4 hrs post-infection with 4mM PBA, 100nM 25(OH)D_3_ (25D) or co-treatment. (B) Expression of proteinase 3 (*PRTN3*) in whole blood (WB), PBMC and MN compared to MDM ± *Mtb* infection ± 25(OH)D_3_ or co-treatment (Co). (C) Treatment of MDM 4 hrs post-infection in the presence or absence of human neutrophil proteinase 3 (PR3). Median ± IQR (A,C) or Mean ± SD (B); n = 5–10; repeated measures 1-way ANOVA with Bonferroni’s multiple comparisons test (A, B) or Wilcoxon matched-pairs test (C): *, P<0.05; **, P<0.01, ns, non-significant.

Finally, as we found a significant synergistic effect of PBA on 25(OH)D_3_ induction on *CAMP* expression (Fig 5B), we investigated whether co-treatment with PBA enhanced 25(OH)D_3_-dependent restriction of *Mtb* growth via cathelicidin production over 7 days of culture, treating macrophages 4 hrs post-infection with either mono- or dual-therapy. Consistent with dose response experiments (Fig 2D), 4mM PBA restricted growth after 7 days (2.8-fold growth, compared to 7.9-fold growth in untreated cells, P = 0.0012), treatment with 25(OH)D_3_ alone resulted in 6.9-fold growth (although not significantly different to control), while co-treatment resulted in 2.4-fold growth (P = 0.0017), and there was no synergistic effect compared to PBA monotherapy (Fig 6A). We also confirmed by checkerboard Alamar Blue assay that there was no direct effect of 25(OH)D_3_ on *in vitro Mtb* growth, in the absence or presence of PBA (S8 Fig).

The fact that there was no significant effect of 25(OH)D_3_ monotherapy or synergistic reduction in *Mtb* growth, despite the 100-fold synergistic induction of *CAMP* expression, suggested that the antimicrobial action of cathelicidin may not have been activated in our *in vitro* model. Activation requires proteolytic processing of the translated pro-LL-37 into active LL-37 by proteinase 3 (PR3) [8]. We therefore investigated expression of *PRTN3* (translated into PR3) in MDM compared to whole blood (WB), PBMC, MN and infected MDM treated ± 25(OH)D_3_ and PBA. We found MDM down-regulated *PRTN3* 6-15-fold (P<0.038) compared to PBMC, WB and MN and that *PRTN3* was further down-regulated over 72hr of infection (Fig 6B). We therefore added PR3 to infected MDM during treatment with 25(OH)D_3_ ± PBA to determine whether LL-37 activation was required for 25(OH)D_3_-mediated *Mtb* growth inhibition. PR3 addition significantly inhibited *Mtb* growth in 25(OH)D_3_-treated MDM to the same level seen with PBA co-treatment, while addition of PR3 during co- treatment further reduced *Mtb* growth (Fig 6C). These findings suggest that 25(OH)D_3_ and PBA restrict *Mtb* growth in MDM via a common cathelicidin-mediated pathway and that PBA also inhibits growth independantly of cathelidicin.

## Discussion

PBA is currently used to treat a range of clinical disorders, due to its pleiotropic effects on cellular differentiation, proliferation and as a prodrug for phenyl acetate. Here we show that clinically attainable concentrations of PBA 1) restrict intracellular and *in vitro* growth of *Mycobacterium tuberculosis*, 2) inhibit *Mtb* phagocytosis by PBMC and MDM, 3) induce antimicrobial and inflammatory pathway genes, including *LTF*, *PROC*, and the inflammasome cascade by MDM and 4) augment 25(OH)D_3_-mediated anti-mycobacterial and anti-inflammatory effects. Our findings suggest that combined PBA and vitamin D may prove an enhanced adjunct therapy to intensive-phase anti-TB treatment; mediated by both broad host-directed and bacterial-directed actions that have the potential to enhance bacterial control as well as resolve lung pathology through broad-ranging anti-inflammatory effects.

PBA has previously been suggested as an antimicrobial agent for the treatment of respiratory infections, due to its ability to reverse the inhibition of expression of the rabbit *CAMP* homologue, *CAP-18*, in lung and trachea epithelium during *Shigella* infection and induce proCAP-18 processing [35, 36]. For the first time we show that PBA not only induces *CAMP* expression in human macrophages during *Mtb* infection, independently and synergistically with physiological concentrations of 25(OH)D_3_, but it also synergistically enhanced expression of the most highly induced vitamin D-regulated gene *CYP24A1* and three genes previously not known to be regulated by vitamin D: *IL36G*, *IL37* and *TREM1* (Fig 4). All three have immunomodulatory properties and therefore may be key regulators of yet unrecognised vitamin D-mediated immune modulation.

Both members of the IL-1 superfamily, IL-36G and IL-37 have opposing effects, being pro- and anti-inflammatory, respectively [37, 38]. We found that *IL36G* was induced by *Mtb* infection while *IL37* was not. Furthermore, both genes were synergistically induced by PBA and 25(OH)D_3_, irrespective of *Mtb* infection, indicating treatment alone was required for induction.

Our observations are the first reported evidence that vitamin D signalling increases *IL37* transcript levels. IL-37 has recently been identified as a natural innate inhibitor, suppressing macrophage TLR-induced cytokine and chemokine secretion of IL-1α (OMIM 147760), IL-1β, IL-6 (OMIM 147620), IL-12, G-CSF (OMIM 138970), GM-CSF (OMIM 138960), and TNF by up to 98%, without regulating IL-10 or IL-1RA (OMIM 147679). Mature IL-37 has been shown to traffic to the nucleus after caspase-1 processing where it complexes with Smad-3 to facilitate TFGβ mediated cytokine suppression, as well as reducing phosphorylation of p38 MAPK, and STAT1-4 [38]. IL-37 may therefore be an additional mediator by which vitamin D elicits its broad anti-inflammatory effects

TREM1 (triggering receptor on myeloid cells-1) regulates inflammation by modulating TLR and NACHT-LRR (NLR) signals [39, 40]. While *TREM1* is constitutively expressed by myeloid cells, the level of induction influences the outcome of infection, such that in a mouse model of sepsis, moderate silencing of *TREM1* improved survival, but near-complete silencing reduced neutrophil oxidative burst and increased mortality [41]. We found that *TREM1* was moderately induced by *Mtb* infection and it was synergistically induced by 25(OH)D_3_ and co-treatment with PBA, irrespective of infection (Figs 4 and S4).

Through the use of MAPK inhibitors we determined that PBA-25(OH)D_3_ synergistic regulation was mediated particularly via p38 or ERK1/2 signalling, but differed by gene, and that the synergistic regulation of *TREM1* was MAPK-independent. We also found that PBA alone induced *VDR* expression, and a recent study in epithelial cells has shown *VDR* knockdown prevents *CAMP* induction by PBA-25(OH)D_3_ co-treatment, indicating *VDR* is vital to this synergistic regulation [42].

Surprisingly, despite the greater than 50-fold induction of *CAMP* expression by 25(OH)D_3_ which was synergistically induced by PBA co-treatment, we found a trend but no significant effect of 25(OH)D_3_ on *Mtb* growth in our MDM model. We found MDM significantly down-regulate *PRTN3* expression and that exogenous PR3 is required to activate 25(OH)D_3_-dependant growth suppression in MDM. *In vivo*, activated neutrophils, recruited by the infected macrophages, could be a source of exogenous PR3. However, lack of vitamin D-mediated antimicrobial activity *in vivo* may be due to unavailability of PR3 to macrophages and future studies should investigate the distribution of PR3 in human TB lung tissues.

We also found that PR3 enhanced growth suppression during co-treatment, which was dominated by PBA in the absence of PR3 to activate cathelicidin (Fig 6). This suggests that the synergistic anti-*Mtb* effects of PBA and 25(OH)D_3_ are likely partially mediated via cathelicidin, but PBA also had cathelicidin-independent effects on *Mtb* growth.

We found that growth restriction by PBA was both MDM-dependent and-independent. Clinically attainable concentrations of PBA completely inhibited *Mtb* growth in broth, with a reduced MIC_99_ under acidic conditions, when *Mtb* was inoculated into PBA-containing medium at low density. Moreover, when high density exponentially growing cultures were treated with PBA, growth was considerably restricted and this growth inhibition was maintained even when PBA was removed, suggesting that PBA may interfere with a structural component required for replication, as the defect was long-lived. This theory is supported by the phenotypic change of PBA-treated bacilli observed by confocal microscopy.

While it may be suggested that the intracellular restriction of *Mtb* growth was due entirely to the direct effect of PBA on *Mtb*, we made a number of observations suggesting otherwise: 1) PBA modulates macrophage function independently of infection within 24–48 hrs; 2) pre-treatment of *Mtb* with PBA did not significantly inhibit 4 hrs uptake or 7 day intracellular growth; 3) significant inhibition of intracellular *Mtb* growth was only observed after 7 days of treatment, whilst growth in broth was inhibited after 2 days. The last observation may also be explained by the *Mtb* target of PBA only being expressed intra-phagosomally after a few days of infection, with acidification of the phagosome potentially enhancing the direct action of PBA. However the fact remains that PBA modifies infected-macrophage function more rapidly than the effect on *Mtb* is observed, suggesting a cell-mediated effect on growth also exists.

Consistent with PBA inhibiting *Mtb* growth via an MDM-dependant mechanism, we found PBA induced expression of *LTF*, encoding the antimicrobial agent and iron chelator lactoferrin. Lactoferrin has been shown to enhance IFNγ-mediated killing of *Mtb* in MDM and oral administration of lactoferrin to *Mtb*-infected mice decreased lung CFU and inflammation [32]. PBA also induced expression of *PROC*, encoding the anti-inflammatory and anti-coagulant Protein C. Activated protein C (aPC) has been shown *in vitro* to suppress expression of the p50 and p52 subunits of NF-κB, blocking NF-κB signalling [33], increasing production of IL-10 and TGFβ [43] and *in* vivo, blocking neutrophil chemotaxis and adhesion to vascular endothelial cells, resulting in decreased lung infiltration of neutrophils in an LPS-induced lung injury model [44]. The recent interest in neutrophils as a primary mediator of pathology in active TB suggests PBA-induced regulation of *PROC* may enhance resolution of pathologic inflammation during TB treatment [45]. The anti-coagulation effect of PROC may also reduce disseminated intravascular coagulation (DIC), which has a high mortality rate in TB patients [46].

Four genes in the inflammasome pathway which may augment the antimicrobial activity of macrophages were also significantly induced by PBA [47]. Surprisingly, despite *CASP1*, *IL1B*, *NCF4* and *NLRP3* expression being induced by PBA, IL-1β secretion was not. However, this may be because IL-37, which is known to inhibit IL-1β secretion [38], was simultaneously induced by PBA. In support of this theory, co-treatment with PBA attenuated the secretion of IL-1β induced by 25(OH)D_3_, at the same time as synergistically inducing *IL37* expression. Further research is required to clearly delineate these interactions.

The role of MAPK signalling in the vitamin D-independent transcriptional effects of PBA was also investigated; although MAPK inhibition partially attenuated expression of *LTF*, *PROC* and inflammasome-associated genes, it did not prevent their PBA-mediated induction. Future work will investigate the role of PBA as a HDACi in regulating the expression of these genes.

Our findings are limited by the fact that we have not yet confirmed the direct and cell-mediated antimicrobial mechanisms of PBA, although we have observations that indicate PBA may be acting on the cell wall. Through the development of PBA-resistant mutants, transposon mutagenesis libraries and macrophage knockdown of *LTF*, future research will more clearly delineate these mechanisms. Moreover, while we investigated the effect of PBA on *Mtb* uptake and growth in PBMC we did not characterise the transcriptional effect of PBA on leukocytes other than macrophages. Recent work has shown that PBA and 1α,25(OH)_2_D_3_ co-treatment of MN during differentiation into dendritic cells (DC) promotes the development of a CD14^+^/CD1a^-^ DC subset which produces enhanced levels of cathelicidin and reactive oxygen species (ROS) and which have enhanced anti-*Staphylococcus aureus* activity [48]. It is therefore likely that vitamin D and PBA have additional cell-mediated effects on the TB immune response, yet to be elucidated.

Together, our results suggest that PBA treatment could independently restrict *Mtb* growth *in vivo* as well as enhance the antimicrobial and anti-inflammatory effects of vitamin D, such that combined PBA and vitamin D therapy during anti-TB treatment may be superior to vitamin D alone. Clinical trials also need to be conducted to evaluate the efficacy of adjunctive PBA in reducing time to culture conversion, independent of vitamin D. However, the combined effect of these two compounds together on anti-microbial and particularly on anti-inflammatory molecules, which are proposed to have a significant benefit on reducing morbidity in TB patients, suggest this combined adjunct therapy might be of greater benefit than either compound alone. The fact that the first Phase 2 trial to investigate the *in vivo* efficacy of this combined therapy was recently completed in Bangladesh, is therefore timely [29]. Our findings of broad and synergistic action of these two compounds will have particular relevance to the interpretation of results from this trial, which will also provide the first *in vivo* study of PBA as an adjunctive therapy for TB.

## Methods

### Ethics statement

Human leukocytes were obtained from healthy volunteers (n = 21). PBMCs from healthy donors were obtained from buffy coats processed by the National Blood Services, Colindale, UK, or heparinised whole blood from healthy volunteers following an explanation of the nature and possible consequences of the study and written informed consent. Ethical approval was received from the Human Research Ethics Review Board of the Faculty of Health Sciences, University of Cape Town, and UK MRC National Institute for Medical Research.

### *Mtb* culture

*Mtb* H37Rv frozen stocks were prepared as previously described [49] (S1 File). For PBA treatment experiments, *Mtb* was inoculated from frozen stocks into 7H9/ADC/0.01% tyloxypol and grown in rolling culture at 37°C. Ten-fold serial dilutions of exponential phase culture were plated on 7H11/OADC agar containing 0-4mM PBA and incubated at 37°C with CFU monitored over 21 days. To analyse the effect of PBA in liquid culture, exponential phase cultures were sub-cultured to OD_600_ 0.01 and after 4 days growth (Average OD_600_ of 0.56) PBA was added to at final concentrations of 0.4-4mM. OD_600_ was monitored over 10 days. Two and six days after addition of PBA, 10-fold serial dilutions (in PBS/0.05% Tween 80) of each culture were plated for CFU analysis. After 10 days of treatment, each rolling culture was pelleted, washed twice in PBS/0.01% tyloxypol and resuspended in medium without PBA to OD_600_ 0.1 and culture continued for a further 8 days, monitoring OD_600_. PBA supplemented media and an aliquot of each 10-day PBA-treated culture was twice filter sterilised and pH was measured (SevenEasy conductivity meter, Mettler Toledo). For the Alamar Blue (Invitrogen) reduction checkerboard assays *Mtb* was inoculated in 96-well plates at OD_600_ 0.0002 in 7H9/ADC containing 2-fold serial dilutions of PBA, 25(OH)D_3_ or HCl. After 13 days of culture, a tenth of the volume of Alamar Blue was added and colour formation observed after overnight incubation.

To determine the effect of PBA pre-treatment of *Mtb* on the outcome of MDM infection, *Mtb* stocks were prepared by treating an exponential phase culture at 0.5 OD_600_ ± 4mM PBA for 4 days. Cultures were pelleted, washed twice in PBS/0.05% and resuspended in 7H9, OD_600_ determined and then an infection stock prepared in RPMI for addition to MDM at final OD_600_ 0.005 (0.2% 7H9). Infection stocks were titrated and plated for CFU, confirming MOI 1:1 (S3 Fig).

For imaging, GFP-H37Rv was grown to exponential phase in 7H9/0.05% Tween 80 with 25μg/ml kanamycin, subcultured to 0.18 OD_600_ and grown ± 4mM PBA for 4 days. 1ml of culture was fixed in 6% PFA for 45min, pelleted, washed in PBS and resuspended in water or Mowiol for mounting under coverslip. Slides were viewed on an Axiovert 200M LSM 510 Meta confocal microscope (Zeiss) and images analysed in Zen Blue 2012 software (Zeiss).

### Culture of PBMC and MDM

PBMC were prepared on a Ficoll-Paque density gradient and used immediately without cryopreservation. PBMC were cultured at 5x10^5^ cells/48-well in RPMI 1640 (containing 10mM sodium pyruvate, 50mM glutamine and 10% foetal calf serum (FCS)) for 3 days prior to infection at 37°C/5% CO_2_. To generate MDM, MN were obtained from PBMC by CD14^+^ magnetic-activated cell sorting (MACS, Miltenyi). MN were cultured at 2x10^6^ cells/6-well in RPMI supplemented with 5ng/ml GM-CSF for 6 days. Following differentiation, MDM were detached and plated at 9x10^4^ cells/96-well in RPMI and rested at 37°C for one hour before infection.

PBMC were infected at a multiplicity of infection (MOI) normalized to average MN count (10% PBMC), giving MOI bacillus:PBMC 0.1:1. At the time of PBMC infection a 2-fold serial dilution of PBA (0-8mM) was added to cultures. After 4 and 96 hrs, PBMC were pelleted by centrifugation, washed twice with PBS, lysed for 30min with H_2_O/0.05% Tween 80 and 5-fold serial dilutions of lysate plated for intracellular CFU.

MDM were infected (MOI 1:1) for 4 hrs, extracellular *Mtb* removed by washing cells twice with PBS and then cultured in RPMI containing a 2-fold serial dilution of PBA (0.5-4mM), a 10-fold serial dilution of 25(OH)D_3_ (10-1000nM), 4mM PBA+100nM 25(OH)D_3_, or vehicle control (0.01% EtOH) for up to 7 days. To determine the effect of PBA on MDM phagocytic function, MDM were treated for 48 hrs with 4mM PBA, washed three times with PBS, infected with *Mtb* and intracellular *Mtb* plated for CFU 4 hrs post-infection.

Additional 25(OH)D_3_ and co-treated cultures were also supplemented with 10μg/ml PR3 at the time of treatment and CFU monitored 7 days post-infection. For MAPK signalling inhibition, MDM were pre-incubated for 1hr with 20μM SB202190, SP600125, U0126 (Sigma) or vehicle control (0.08% DMSO), prior to and during stimulation.

### Flow cytometry

MDM were detached after 48 hrs treatment with PBA, by incubating in Acutase (Invitrogen) at 37°C for 30min, washed twice with PBS, and suspended in PBS/5% FCS. Cells were incubated with either monoclonal mouse anti-human CD35/FITC, CD11c/PE, CD16/PE.Cy5, CD11b/APC conjugated antibodies or respective IgG-conjugated antibodies (BD Bioscience) in the dark at 4°C for 30 min. Cells were washed, resuspended in CytoFix/Perm (BD Bioscience) for 20 min at 4°C and then washed twice and resuspended in PBS/5% FCS. An aliquot of unfixed cells were also incubated with propidium iodide. Ten thousand events were analysed for each sample by immunofluorescence using flow cytometry (BD FACSCalibur). Results were analysed using Flow Jo (version 10).

### RT-PCR arrays

RNA was extracted from MDM samples following homogenisation in TRI Reagent (Sigma-Aldrich) and storage at -80°C until batch-processed. RNA was extracted with chloroform in phase lock tubes (5 Prime) following the manufactures protocol, using linear poly-acrylamide (Ambion) as a carrier protein and suspension in RNase-free H_2_O (Sigma). RNA integrity and concentration was determined on a NanoDrop 2000 (Thermo Scientific) and by running on a 1.5% TAE agarose gel. 150ng RNA was reverse transcribed using the RT^2^ First Strand Kit (SABiosciences) which includes a genomic DNA (gDNA) elimination step. cDNA was analysed using 384-well RT^2^ Profiler Custom PCR Arrays (SABiosciences) which included 28 genes involved in innate and adaptive immunity (*CAMP*, *CASP1*, *CXCL7*, *CXCL8*, *CXCL9*, *CXCL10*, *IFNB1* (OMIM 147640), *IFNG*, *IL1B*, *IL6*, *IL10*, *IL12A*, *IL12B* (OMIM 161561), *IL23A* (OMIM 605580), *IL36G*, *IL37*, *LTF*, *NOD2*, *NCF4*, *NLRP3*, *PGLYRP1* (OMIM 604963), *PROC*, *ARG1*, *TNF*, *TREM1*) and vitamin D metabolism (*CYP24A1*, *CYP27B1*, *VDR*), plus the house-keeping (HK) gene *RPL13A* (UniProt P40429) and three control reactions to monitor gDNA contamination and reverse transcription efficiency on the Roche 480 platform. A comparison of 5 HK genes across all treatment and infection conditions indicated that *RPL13A* was the most stable (S9 Fig). Fold induction was calculated from Ct values using the ΔΔCt method normalising all samples to baseline untreated.

### RT-PCR

For *PRTN3* expression analysis RNA was extracted from 1 million PBMC and MN following homogenisation in TRI Reagent (as for MDM) and from 3ml whole blood collected in Tempus tubes and extracted with the Tempus Spin RNA Isolation Kit (Life Technologies) and storage at -80°C. 100ng RNA was reverse transcribed using SuperScript VILO cDNA synthesis kit (Life Technologies) and real-time RT-PCR was performed using primers (S1 Table) for *PRTN3* and *GNB2L1* (OMIM 176981, HK) with Fast SYBR Green chemistry using 10% cDNA on a QuantStudio 7 (Life Technologies). Stable expression of *GNB2L1* across all sample types was confirmed by Ct comparison (S10 Fig). Expression levels of *PRTN3* were normalised to that of *GNB2L1* by ΔΔCt and fold change to mean MDM expression calculated.

For *CAMP*, *CYP27B1* and *CYP24A1* expression analysis (primer sequences in S1 Table) 100ng RNA from MDM was reverse transcribed using High Capacity cDNA synthesis kit (Life Technologies) and real-time RT-PCR was performed with Fast SYBR Green chemistry), using 10% cDNA on an ABI 7500 fast (Life Technologies). Absolute quantification was carried out using standard curves generated by serial dilution of target amplicon-containing plasmids (pGEM-T easy, Promega), to cover up to 5 logs of amplicon copy number per microliter and absolute copy number normalized to *GNB2L1*.

### Luminex

Supernatants were harvested from MDM infection experiments 24 and 72 hrs post-infection and double sterilized through 0.2uM PVDF filters (Corning). Concentrations of IL-1β, IL-1RA, IL-2 (OMIM 147680), IL-2R, IL-4 (OMIM 147780), IL-5 (OMIM 147850), IL-6, IL-7 (OMIM 146660), IL-10, IL-12 (p40/p70), IL-13 (OMIM 147683), IL-15, IL-17 (OMIM 603149), G-CSF, GM-CSF, IFNα, IFNγ, TNF, CXCL8, CXCL9, CXCL10, CCL2, CCL3, CCL4, CCL5, CCL11 (OMIM 601156), EGF (OMIM 131530), FGFβ (OMIM 134920), HGF (OMIM 142409) and VEGF (OMIM 192240) were quantified using a human 30-plex bead immunoassay panel (Invitrogen) and analysed on a BioPlex 200 platform (Bio-Rad). Analytes at the limit of detection were given the value zero. Concentrations from infected samples were baseline corrected by subtracting the concentration of uninfected samples prior to statistical analysis.

### Statistical analysis

Cells from all donors received all treatments (paired analyses) and RNA, culture supernatants and CFU where matched for donor where multiple analyses where conducted under the same infection conditions, where possible. Infection experiments were performed on up to 10 donors, in triplicate for CFU and in duplicate for RNA and secreted protein analysis. *Mtb* culture experiments were done in duplicate and repeated. Samples were assigned a numerical de-identifier prior to analysis and decoded after raw data was captured. Statistical significance was tested using paired t-test of single treatment analysis, Wilcoxon match-pairs for non-parametric data, 1way-ANOVA, with Bonferroni multiple comparison test for dose response analysis at a single time point or 2way-ANOVA, with Dunnett's multiple comparison test for dose response analysis over multiple time points. GLM, PCA and hierarchical clustering were conducted on gene expression and protein secretion data using Qlucore Omics Explorer 2.2 (Qlucore AB, Lund, Sweden) (S1 File).

## Supporting Information

S1 File Materials and methods. *Mtb* Stock Preparation, treatments and statistical analysis.
(DOCX)

S1 Fig Multiple examples of confocal images of *Mtb* strain GFP-H37Rv treated with and without PBA. (A) *Mtb* treated with PBA are often found bound along the entire shaft of neighbouring bacilli, forming lined-up clusters, unlike untreated cultures (B) which had single bacilli or overlapping bacilli when in clusters. GFP-expression is in green, and phase contrast image in grey, with overlaid images on the right.
(TIF)

S2 Fig Effect of PBA on the pH of culture medium (7H9 or RPMI) before or after *Mtb* infection. There was no effect of PBA on the pH of any medium.
(TIF)

S3 Fig Colony forming units (CFU) of infection stock made from *Mtb* treated with/without 4mM PBA for 4 days prior to infection and 4 hrs uptake by macrophages. There was no difference in CFU of the infection stock (A) or uptake by macrophages (B). Mean ± SD, n = 3 (A) and n = 4 donors, in triplicate (B); Paired t-test, all non-significant.
(TIFF)

S4 Fig MDM gene expression in response to treatment with PBA serial dilution. During *Mtb* infection of MDM PBA regulated gene expression in a dose-dependant manner, mean, n = 2.
(TIF)

S5 Fig MDM secretion in response to treatment with PBA serial dilution. During *Mtb* infection PBA regulated MDM secretion in a dose-dependant manner. Secretion is normalised by subtracting uninfected samples. Mean ± SD; n = 2; 1way-ANOVA, with Dunnett’s multiple testing; *, P<0.05; **, P<0.01.
(TIF)

S6 Fig MDM expression in response to treatment with 25(OH)D_3_ serial dilution. Expression of vitamin D regulated genes *CAMP*, *CYP24A1* and *CYP27B1* by uninfected MDM and cells infected with *Mtb* H37Rv, for 4 hrs prior to treatment, Mean ± SD, n = 2. Differences between samples were analysed for log_10_ transformed data by repeated-measured 2way ANOVA, with Dunnett’s multiple comparison test; *, P < 0.05; **, P < 0.01; ***, P < 0.001; ****, P < 0.0001.
(TIFF)

S7 Fig Uninfected MDM gene expression in response to treatment with PBA and 25(OH)D_3_. PBA + 25(OH)D_3_ synergistically induced expression of *CYP24A1*, *CAMP*, *IL36G*, *IL37* and *TREM1*, while inhibiting expression of *CYP27B1* in uninfected MDM treated 4hrs after plating. *CXCL7*, *CXCL9* and *CXCL10* were not significantly regulated in uninfected MDM. Untreated control (black); 4mM PBA (blue); 100nM 25(OH)D_3_ (red); co-treatment (purple). Mean ± SD, n = 3, in duplicate.
(TIF)

S8 Fig Checkerboard Alamar Blue reduction assay measuring MIC_99_ of 25(OH)D3 in combination with PBA. Two-fold serial dilutions of 25(OH)D (800nM-3.1nM) and for PBA (4mM-0.13mM) showed that 25(OH)D_3_ does not affect *Mtb* growth in this physiologial range and has no effect on growth restiction by PBA. (A) 13 days growth and (B) 14 days growth, 16 hrs after addition of Alamar Blue. Growth occurs in pink wells with visible white *Mtb* pellets, and no growth in blue wells. Red square indicates 100nM 25(OH)D_3_, the concentration used during macrophage experiments.
(TIF)

S9 Fig Analysis of the expression of house keeping (HK) genes in monocyte-derived macrophages. MDM were ± *Mtb* infection, 24 hrs or 72 hrs post-infection ± 4mM PBA and ± 100nM 25(OH)D_3_ treatment. *RPL13A* was the most stably expressed across all conditions. 150ng RNA was added to each cDNA reaction. Expression is represented as cycle threshold (Ct), line indicates median ± IQR, n = 34.
(TIF)

S10 Fig Analysis of the gene expression cycle time (Ct) of *PRTN3* vs the house keeping gene *GNB2L1*. Comparison between whole blood (red), PBMC (dark pink) and monocytes (light pink) vs MDM ± *Mtb* infection for 4, 24 or 72 hrs (black). Line indicates median, n = 3–5 donors.
(TIFF)

S1 Table Primers used for real-time RT-PCR. (XLSX)
