# Supplementary material for: Phenylbutyrate Is Bacteriostatic against Mycobacterium tuberculosis and Regulates the Macrophage Response to Infection, Synergistically with 25-Hydroxy-Vitamin D₃
Source: PLoS Pathog. 2015 Jul 2;11(7):e1005007. doi: 10.1371/journal.ppat.1005007 (PMC4489717; doi:10.1371/journal.ppat.1005007)
Supplement: S1 File — Mtb Stock Preparation, treatments and statistical analysis. (DOCX) [file ppat.1005007.s001.docx]

**Materials and Methods**

***Mtb* Stock Preparation**

*Mtb* frozen stocks were prepared by inoculating a single colony of *Mtb* in 7H9/ADC (BD Biosciences)/0.05% Tween 80 (Sigma) and grown in static culture at 37^o^C for 10 days, then sub-cultured 1/100 in the same medium in the absence of Tween 80 for a further 10 days. Cultures were pelleted, supernatant removed and pellets vigorously shaken with 2-3 mm glass beads for one minute. PBS was added to dispersed *Mtb*, which was again pelleted and the top half of the supernatant, containing single cell *Mtb*, was resuspended with a final volume of 5% glycerol and frozen at -80^o^C. Before and after freezing, stocks were titrated and plated on 7H11/OADC (BD Biosciences) for CFU determination.

**Treatments**

A stock solution of PBA (Calbiochem) was dissolved to a concentration of 1M in H_2_O and stored at -20^o^C. A stock solution of 25(OH)D_3_ was dissolved in 95% ethanol to a concentration of 1mM and stored under argon at -20^o^C. GM-CSF (ProSci) was resuspended in RPMI/5%FCS at 100μM and stored at -20^o^C. Human Neutrophil Proteinase 3 (Calbiochem) was stored at a stock concentration of 0.6mg/ml at -20^o^C. SB202190 (20mg/ml), SP600125 (10mg/ml), and U0126 (10mg/ml) (Sigma) were resuspened in DMSO to stock concentration and stored at 4^o^C. Working dilutions were prepared by adding serial dilutions to media and control samples were prepared using corresponding final concentrations of 0.01% ethanol and/or 0.08% dimethyl sulfoxide.

**Statistical Analysis**

For GLM analysis in Qlucore Omics Explorer, analyte levels were log_2_ converted and normalised to the mean for each analyte and the variance was normalized to 1. For analytes that were undetectable in at least one sample, the ‘limit of detection’ value was added to every measured value for that analyte prior to log2 conversion. Comparisons between 2 treatment groups were conducted using a t-test for GLM, with statistical adjustment for covariates, using the eliminated factors approach, including ‘subject ID’ to adjust for donor variation and ‘time of treatment’ when data from multiple time points were combined for analysis, as previously described [[1](#_ENREF_1)]. Eliminated factors fits a multiple regression model to all covariates, and subtracts the expression values predicted by this model from the observed values in order to remove covariate effects between patients [[2](#_ENREF_2)]. Thresholds of 0.05 for p-values and 0.1 for q-values, which define the lowest false discovery rate (FDR) for which the hypothesis would be accepted under the Benjamini-Hochberg procedure for multiple testing correction, were applied throughout [[3](#_ENREF_3)]. PCA and hierarchical clustering was conducted to visualise the relationships between treatment groups and the effect of ‘infection status’ and ‘time of treatment’ on response to treatment for genes identified to be statistically significant by GML analyses.

**References**

1. Coussens AK, Wilkinson RJ, Nikolayevskyy V, Elkington PT, Hanifa Y, Islam K, et al. Ethnic variation in inflammatory profile in tuberculosis. PLoS Pathogens. 2013;9(7):e1003468. Epub 2013/07/16. doi: 10.1371/journal.ppat.1003468. PubMed PMID: 23853590.

2. Wichura MJ. The Coordinate-Free Approach to Linear Models. Cambridge: Cambridge Univ. Press; 2006.

3. Benjamini Y, Hochberg Y. Controlling the False Discovery Rate - a Practical and Powerful Approach to Multiple Testing. Journal of the Royal Statistical Society Series B-Methodological. 1995;57(1):289-300. PubMed PMID: ISI:A1995QE45300017.
