# Supplementary figures and images for: Phenylbutyrate Is Bacteriostatic against Mycobacterium tuberculosis and Regulates the Macrophage Response to Infection, Synergistically with 25-Hydroxy-Vitamin D₃
Source: PLoS Pathog. 2015 Jul 2;11(7):e1005007. doi: 10.1371/journal.ppat.1005007 (PMC4489717; doi:10.1371/journal.ppat.1005007)

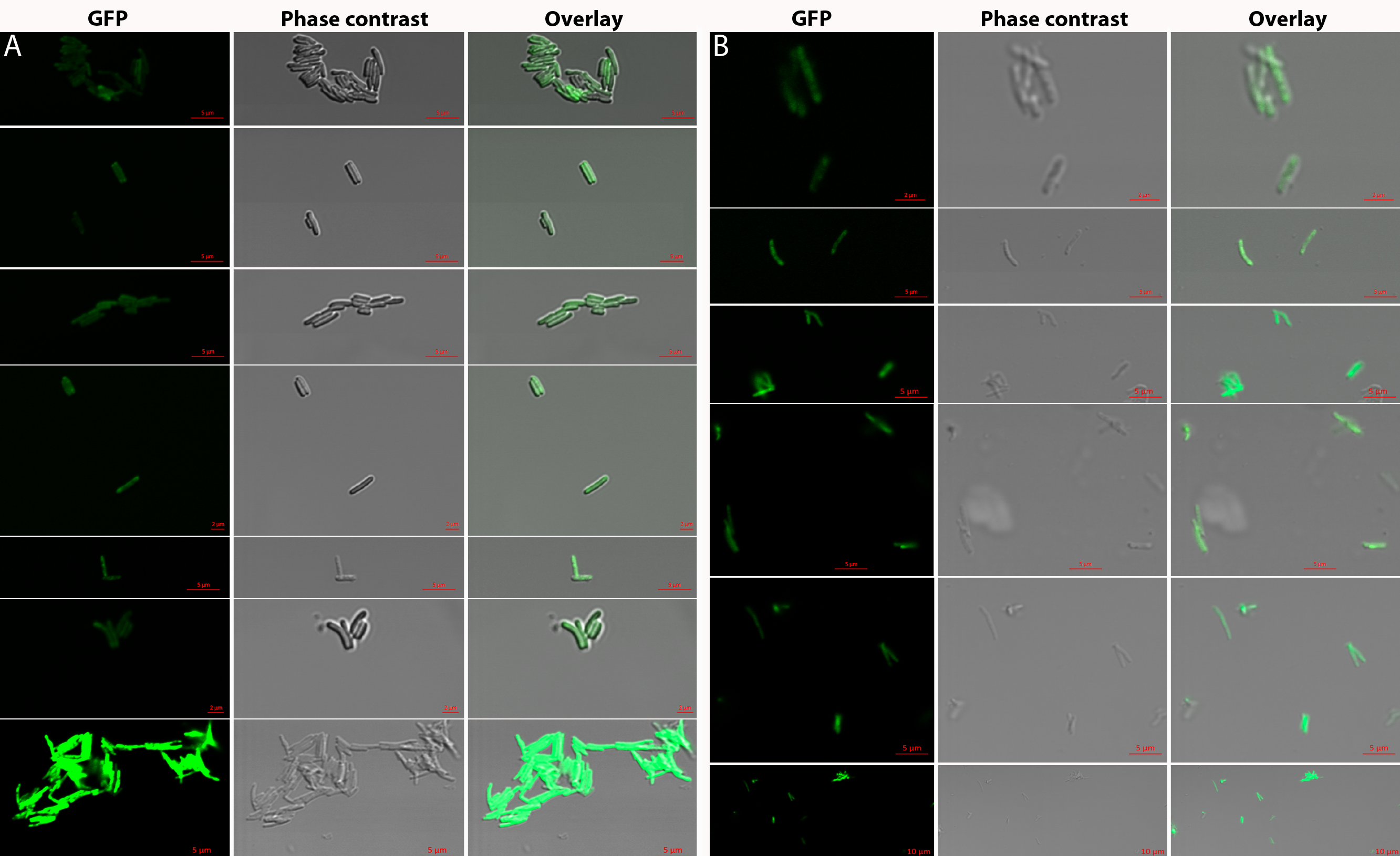

Supplement: S1 Fig — (A) Mtb treated with PBA are often found bound along the entire shaft of neighbouring bacilli, forming lined-up clusters, unlike untreated cultures (B) which had single bacilli or overlapping bacilli when in clusters. GFP-expression is in green, and phase contrast image in grey, with overlaid images on the right. (TIF) [file ppat.1005007.s002.tif]

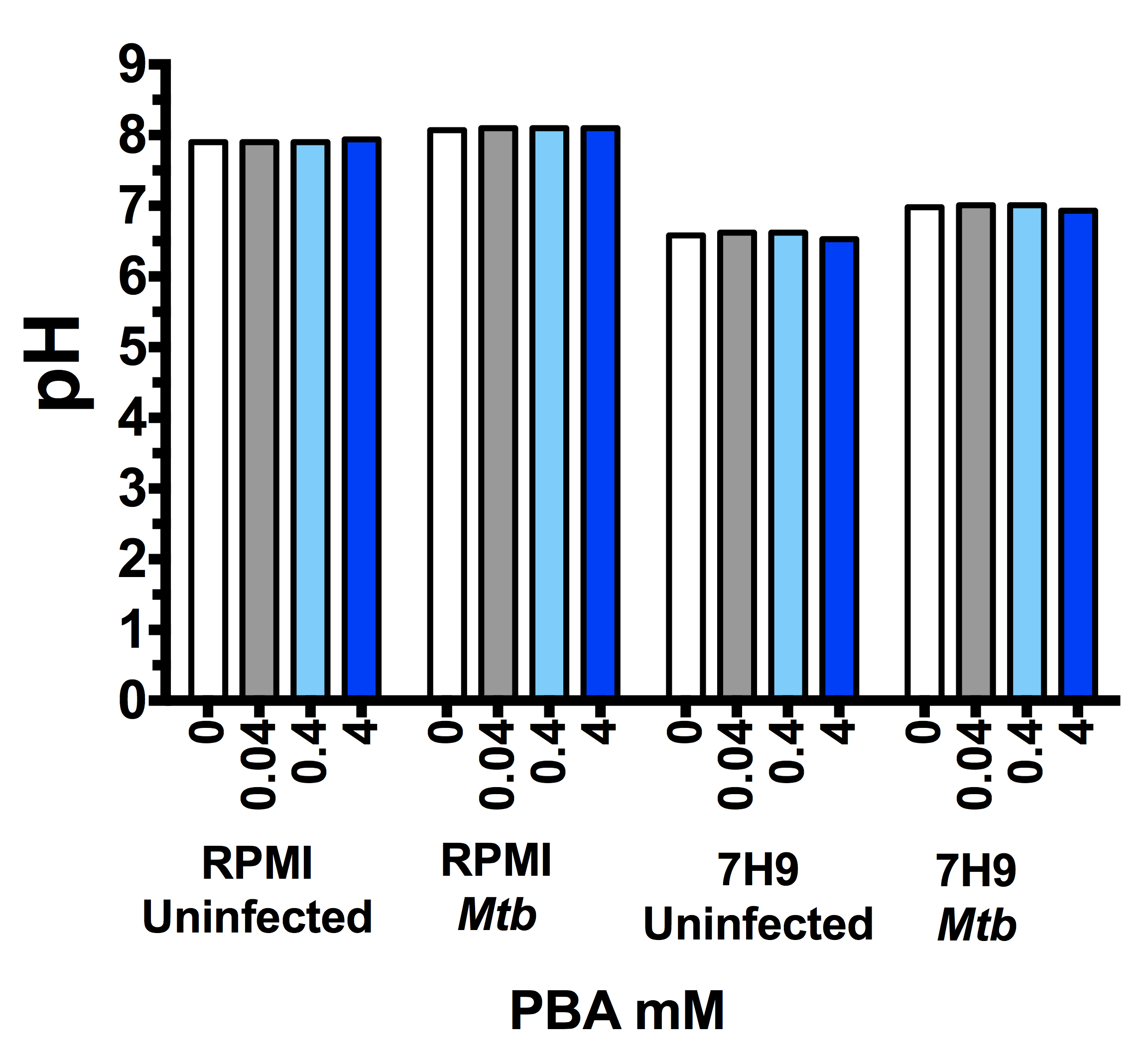

Supplement: S2 Fig — There was no effect of PBA on the pH of any medium. (TIF) [file ppat.1005007.s003.tif]

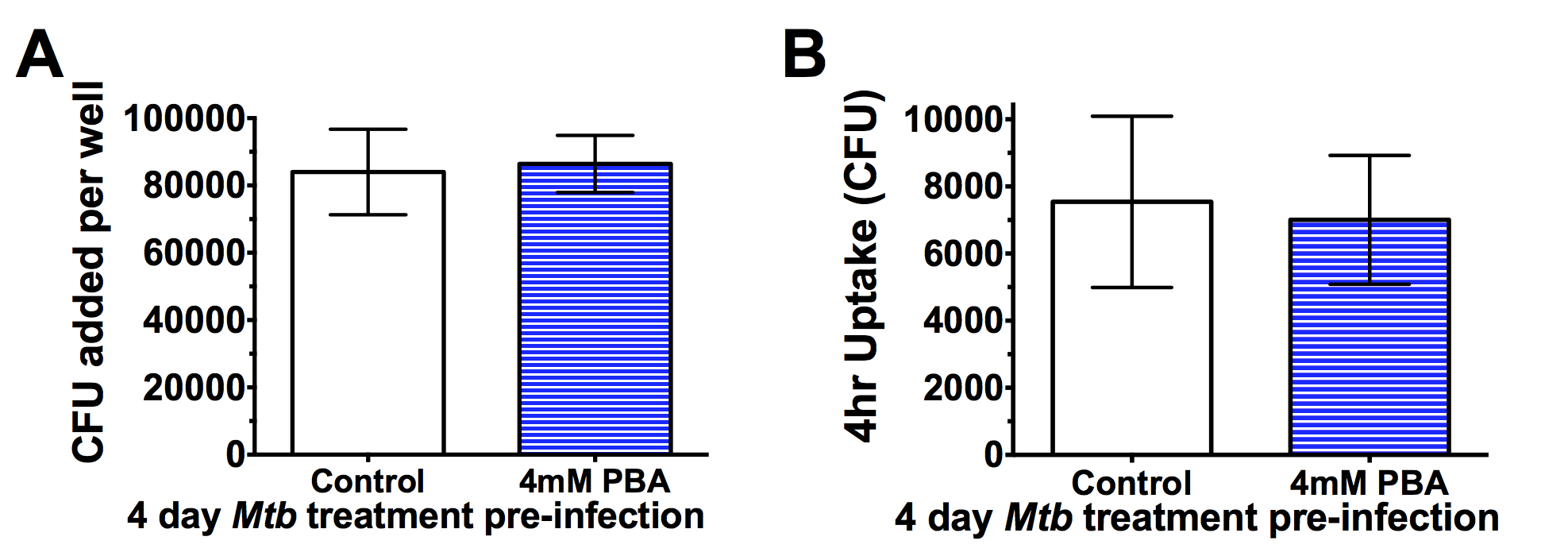

Supplement: S3 Fig — There was no difference in CFU of the infection stock (A) or uptake by macrophages (B). Mean ± SD, n = 3 (A) and n = 4 donors, in triplicate (B); Paired t-test, all non-significant. (TIFF) [file ppat.1005007.s004.tiff]

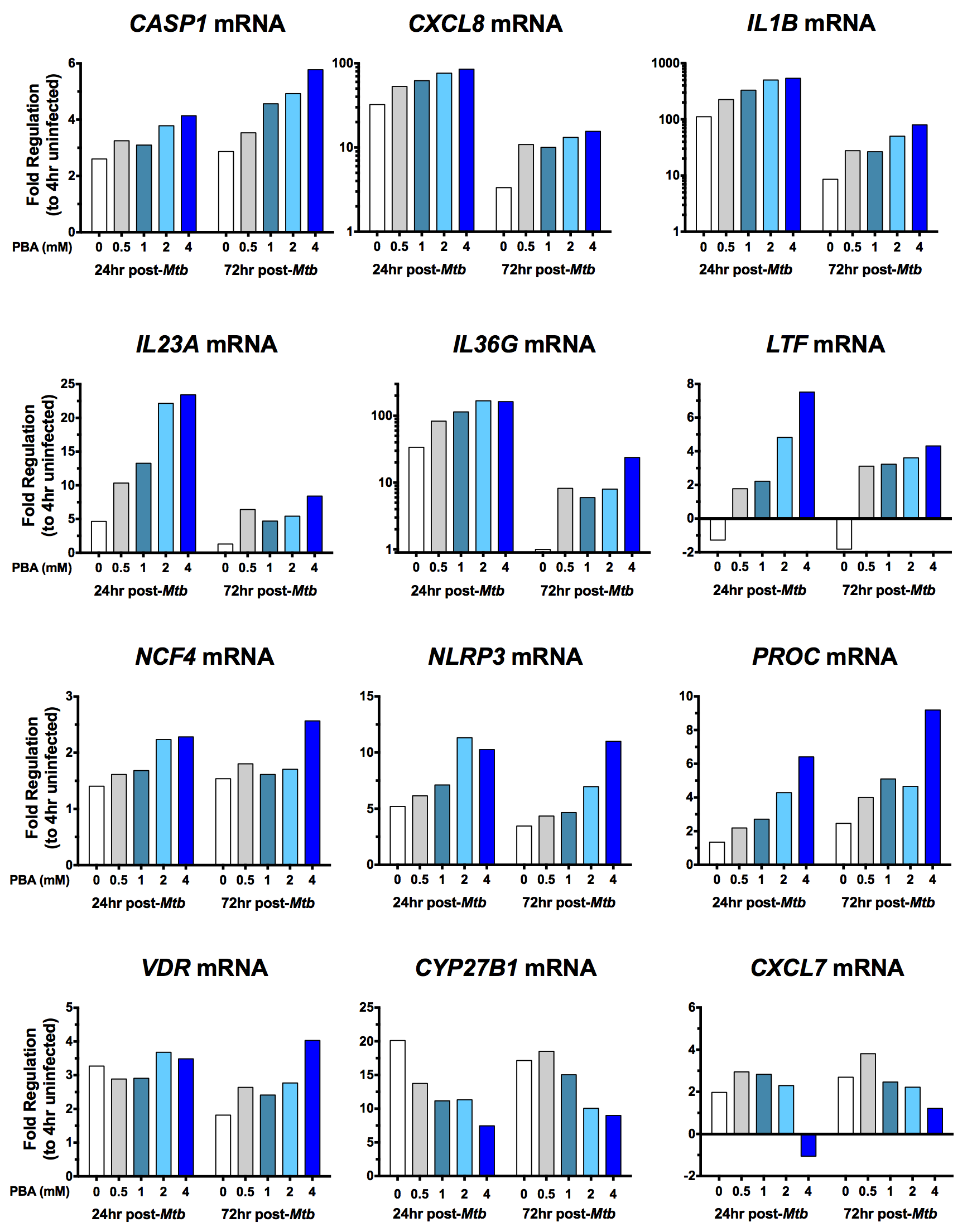

Supplement: S4 Fig — During Mtb infection of MDM PBA regulated gene expression in a dose-dependant manner, mean, n = 2. (TIF) [file ppat.1005007.s005.tif]

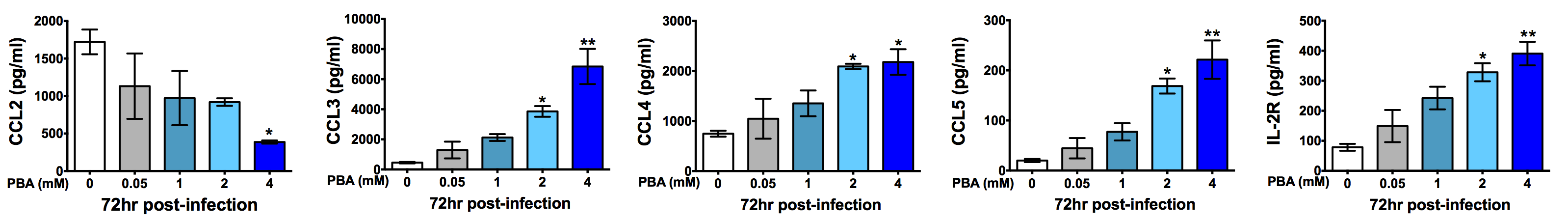

Supplement: S5 Fig — During Mtb infection PBA regulated MDM secretion in a dose-dependant manner. Secretion is normalised by subtracting uninfected samples. Mean ± SD; n = 2; 1way-ANOVA, with Dunnett’s multiple testing; *, P<0.05; **, P<0.01. (TIF) [file ppat.1005007.s006.tif]

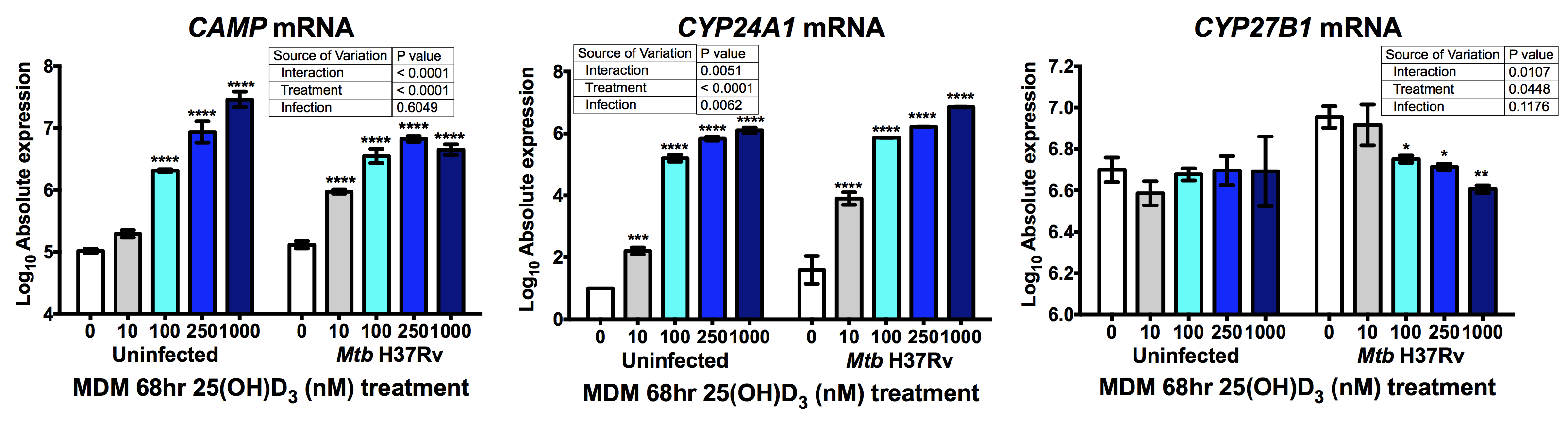

Supplement: S6 Fig — Expression of vitamin D regulated genes CAMP, CYP24A1 and CYP27B1 by uninfected MDM and cells infected with Mtb H37Rv, for 4 hrs prior to treatment, Mean ± SD, n = 2. Differences between samples were analysed for log10 transformed data by repeated-measured 2way ANOVA, with Dunnett’s multiple comparison test; *, P < 0.05; **, P < 0.01; ***, P < 0.001; ****, P < 0.0001. (TIFF) [file ppat.1005007.s007.tiff]

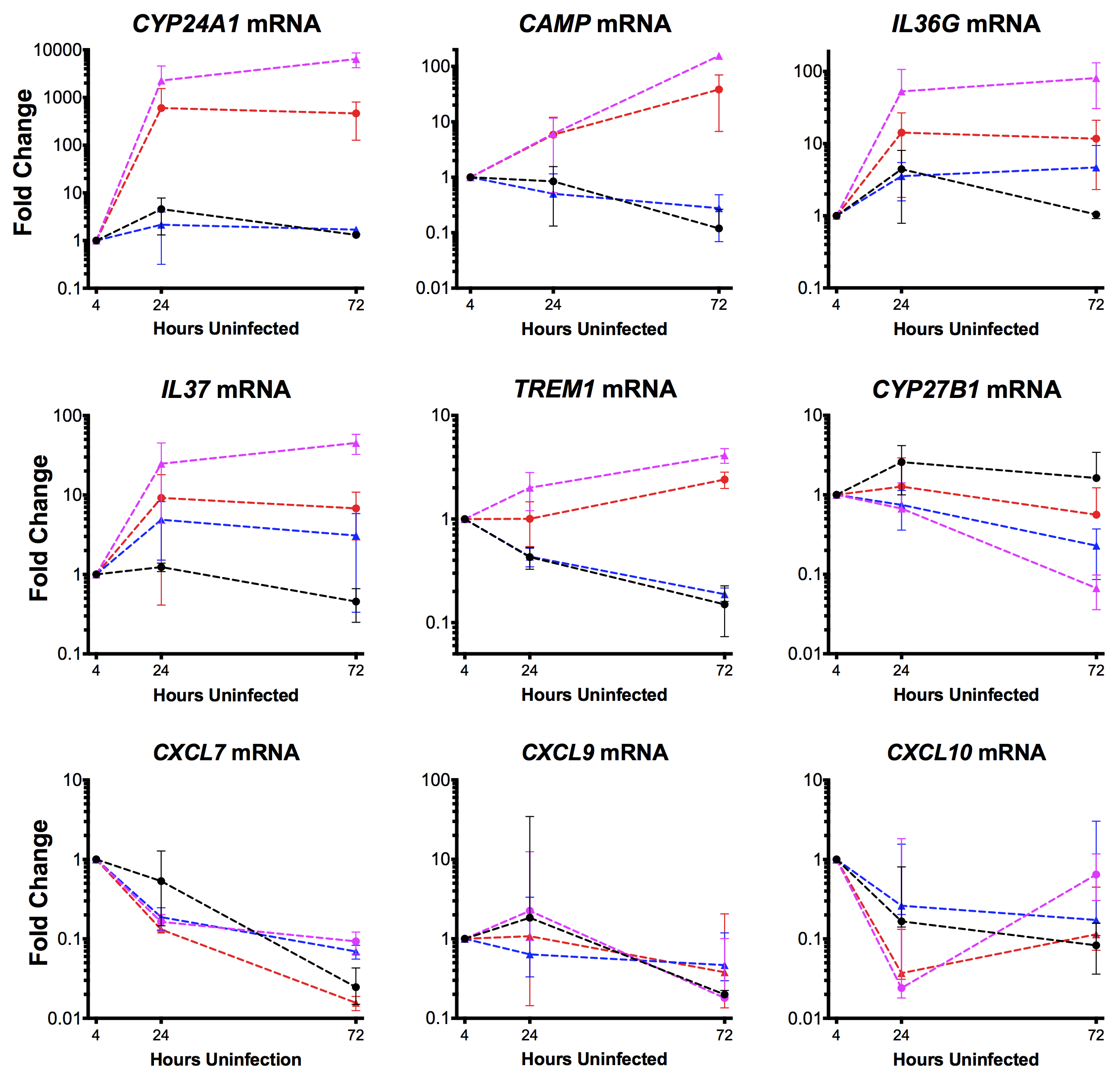

Supplement: S7 Fig — PBA + 25(OH)D3 synergistically induced expression of CYP24A1, CAMP, IL36G, IL37 and TREM1, while inhibiting expression of CYP27B1 in uninfected MDM treated 4hrs after plating. CXCL7, CXCL9 and CXCL10 were not significantly regulated in uninfected MDM. Untreated control (black); 4mM PBA (blue); 100nM 25(OH)D3 (red); co-treatment (purple). Mean ± SD, n = 3, in duplicate. (TIF) [file ppat.1005007.s008.tif]

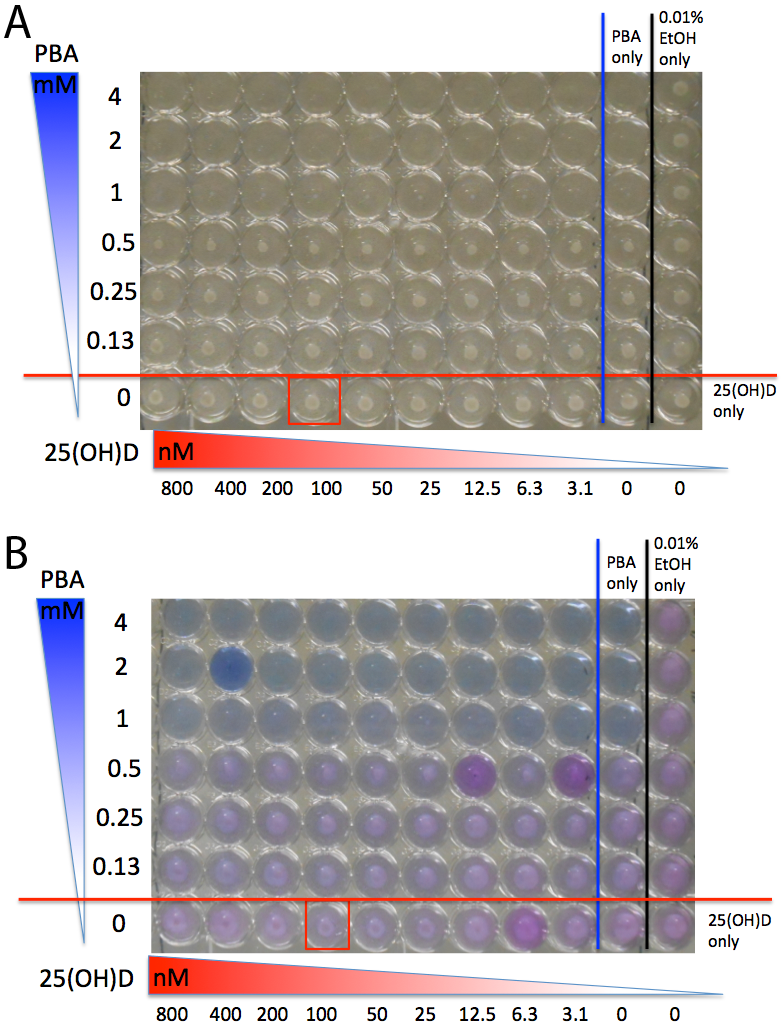

Supplement: S8 Fig — Two-fold serial dilutions of 25(OH)D (800nM-3.1nM) and for PBA (4mM-0.13mM) showed that 25(OH)D3 does not affect Mtb growth in this physiologial range and has no effect on growth restiction by PBA. (A) 13 days growth and (B) 14 days growth, 16 hrs after addition of Alamar Blue. Growth occurs in pink wells with visible white Mtb pellets, and no growth in blue wells. Red square indicates 100nM 25(OH)D3, the concentration used during macrophage experiments. (TIF) [file ppat.1005007.s009.tif]

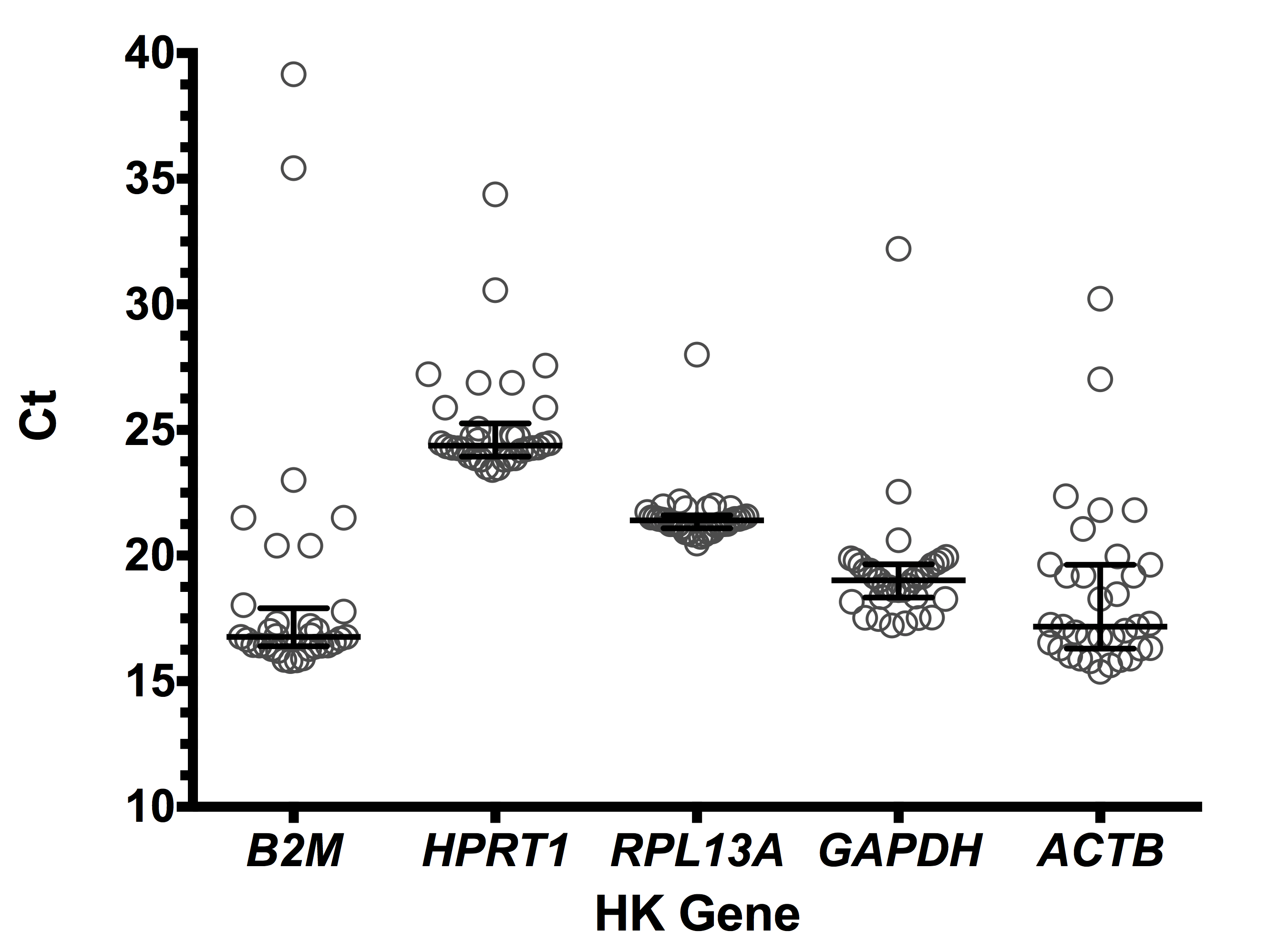

Supplement: S9 Fig — MDM were ± Mtb infection, 24 hrs or 72 hrs post-infection ± 4mM PBA and ± 100nM 25(OH)D3 treatment. RPL13A was the most stably expressed across all conditions. 150ng RNA was added to each cDNA reaction. Expression is represented as cycle threshold (Ct), line indicates median ± IQR, n = 34. (TIF) [file ppat.1005007.s010.tif]

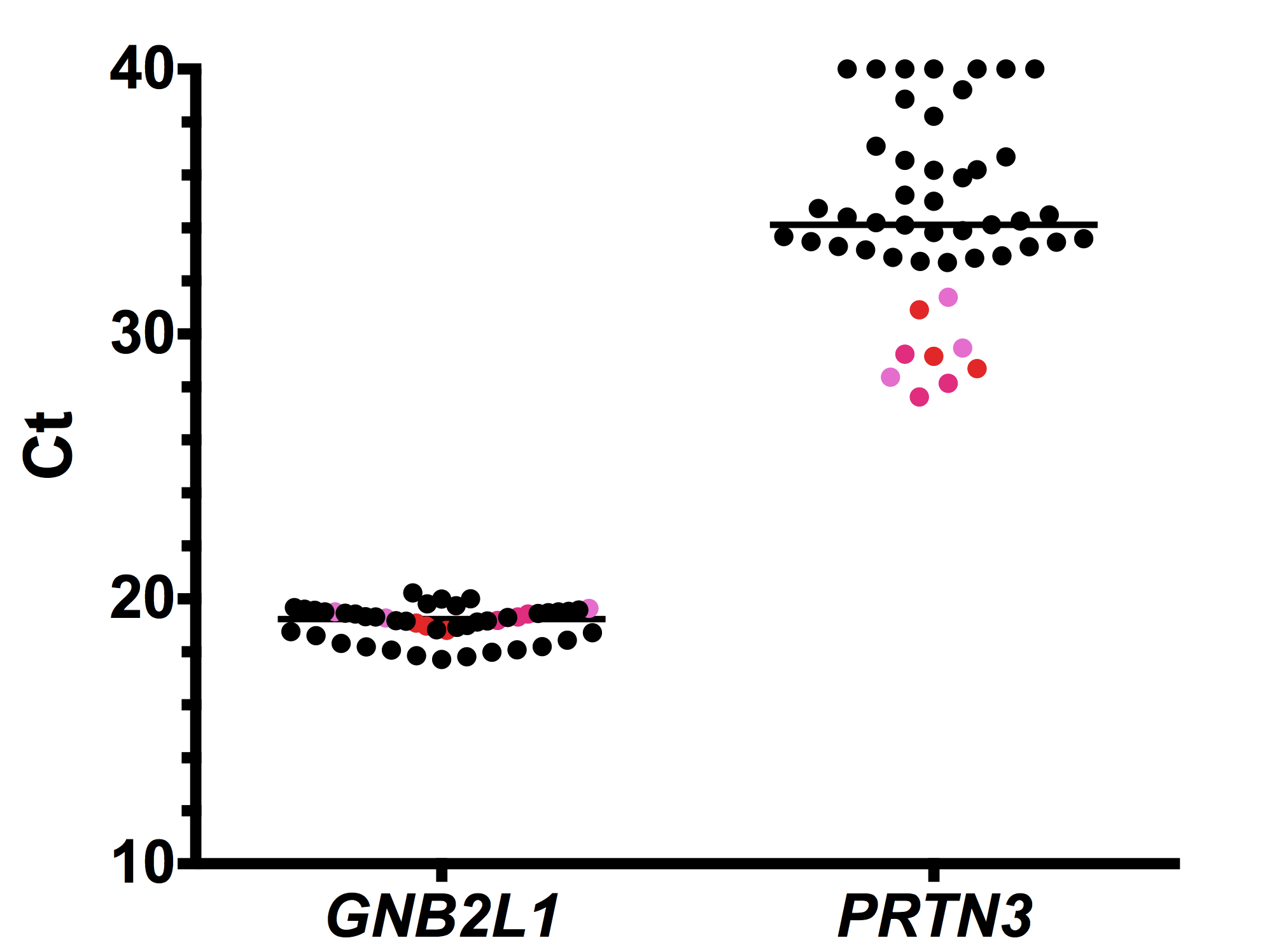

Supplement: S10 Fig — Comparison between whole blood (red), PBMC (dark pink) and monocytes (light pink) vs MDM ± Mtb infection for 4, 24 or 72 hrs (black). Line indicates median, n = 3–5 donors. (TIFF) [file ppat.1005007.s011.tiff]
